# Targeting the ALK–CDK9-Tyr19 kinase cascade sensitizes ovarian and breast tumors to PARP inhibition via destabilization of the P-TEFb complex

**DOI:** 10.1038/s43018-022-00438-2

**Published:** 2022-10-17

**Authors:** Yu-Yi Chu, Mei-Kuang Chen, Yongkun Wei, Heng-Huan Lee, Weiya Xia, Ying-Nai Wang, Clinton Yam, Jennifer L. Hsu, Hung-Ling Wang, Wei-Chao Chang, Hirohito Yamaguchi, Zhou Jiang, Chunxiao Liu, Ching-Fei Li, Lei Nie, Li-Chuan Chan, Yuan Gao, Shao-Chun Wang, Jinsong Liu, Shannon N. Westin, Sanghoon Lee, Anil K. Sood, Liuqing Yang, Gabriel N. Hortobagyi, Dihua Yu, Mien-Chie Hung

**Affiliations:** 1 Department of Molecular and Cellular Oncology, The University of Texas MD Anderson Cancer Center, Houston, TX USA; 2 UT Health Graduate School of Biomedical Sciences, The University of Texas MD Anderson Cancer Center, Houston, TX USA; 3 Graduate Institute of Biomedical Sciences, Research Center for Cancer Biology, and Center for Molecular Medicine, China Medical University, Taichung, Taiwan; 4 Department of Breast Medical Oncology, The University of Texas MD Anderson Cancer Center, Houston, TX USA; 5 Department of General Surgery, Xinhua Hospital Affiliated to Shanghai Jiao Tong University School of Medicine, Shanghai, China; 6 Department of Anatomic Pathology, Division of Pathology and Laboratory Medicine, The University of Texas MD Anderson Cancer Center, Houston, TX USA; 7 Department of Gynecologic Oncology and Reproductive Medicine, The University of Texas MD Anderson Cancer Center, Houston, TX USA; 8 Department of Systems Biology, Division of Basic Science Research, The University of Texas MD Anderson Cancer Center, Houston, TX USA; 9 Center for RNA Interference and Non-Coding RNA, The University of Texas MD Anderson Cancer Center, Houston, TX USA; 10 Department of Biotechnology, Asia University, Taichung, Taiwan

**Keywords:** Molecular biology, Cancer, Biomarkers

## Abstract

Poly(ADP-ribose) polymerase (PARP) inhibitors have demonstrated promising clinical activity in multiple cancers. However, resistance to PARP inhibitors remains a substantial clinical challenge. In the present study, we report that anaplastic lymphoma kinase (ALK) directly phosphorylates CDK9 at tyrosine-19 to promote homologous recombination (HR) repair and PARP inhibitor resistance. Phospho-CDK9-Tyr19 increases its kinase activity and nuclear localization to stabilize positive transcriptional elongation factor b and activate polymerase II-dependent transcription of HR-repair genes. Conversely, ALK inhibition increases ubiquitination and degradation of CDK9 by Skp2, an E3 ligase. Notably, combination of US Food and Drug Administration-approved ALK and PARP inhibitors markedly reduce tumor growth and improve survival of mice in PARP inhibitor-/platinum-resistant tumor xenograft models. Using human tumor biospecimens, we further demonstrate that phosphorylated ALK (p-ALK) expression is associated with resistance to PARP inhibitors and positively correlated with p-Tyr19-CDK9 expression. Together, our findings support a biomarker-driven, combinatorial treatment strategy involving ALK and PARP inhibitors to induce synthetic lethality in PARP inhibitor-/platinum-resistant tumors with high p-ALK–p-Tyr19-CDK9 expression.

## Abstract (web summary)

Hung and colleagues show that ALK-mediated CDK9 activation increases homologous recombination repair and PARP inhibition resistance and propose combinatorial inhibition of ALK and PARP as therapeutically beneficial for treatment-resistant tumors.

## Main

Platinum-based chemotherapy is frequently used as part of the standard of care for patients with ovarian and breast cancers. However, resistance to platinum compounds occurs frequently and portends a poor prognosis^1,2^. Advances in molecular medicine have led to the development of PARP inhibitors, which have shown great promise in the treatment of a substantial population of patients with ovarian and breast cancer, offering notable benefit over conventional chemotherapy^3,4^. Inhibition of PARP, a key enzyme involved in the repair of DNA single-strand breaks (SSBs) in eukaryotic cells, leads to the accumulation of SSBs, which in turn increases DNA double-strand breaks (DSBs)^5^. In addition to regulating the repair of SSBs, enzymatic activity of PARP at stalled replication forks is critical for stabilization and resumption of DNA replication^6–8^. Furthermore, previous studies have demonstrated that PARP inhibitors not only inhibit the enzymatic activity of PARP but also cause trapping of PARP on DNA, thereby inducing replication fork collapse and formation of DSBs^9–12^. Repair of DSBs is largely dependent on HR and regulated by tumor suppressors, *BRCA1* and *BRCA2* (refs. ^13,14^). Thus, inhibition of PARP in cells with pathogenic loss-of-function mutations in *BRCA1* and *BRCA2* (*BRCA1*/*2*) results in synthetic lethality due to the overwhelming accumulation of DSBs^15,16^. Notably, mutations in *BRCA1*/*2* have been implicated in the pathogenesis of many cancers, such as breast and ovarian cancers^17^, and several PARP inhibitors have been approved by the US Food and Drug Administration (FDA) for *BRCA1*/*2*-mutant ovarian and breast cancers^3,4^. However, despite the strong scientific rationale and impressive prolongation of progression-free survival with maintenance PARP inhibitor monotherapy in patients with platinum-sensitive, *BRCA1*/*2*-mutant ovarian cancers^18,19^, acquired resistance to PARP inhibitors and the limited therapeutic efficacy of PARP inhibitors in patients with platinum-resistant cancers remain a substantial clinical challenge^20^. Thus, a deeper understanding of the mechanisms underlying PARP inhibitor resistance is critical to improve patient outcomes by identifying new biomarkers that can effectively predict response to PARP inhibitors and elucidating new molecular targets that can be exploited in the development of combinatorial treatment strategies.

Aberrant activation of receptor tyrosine kinases (RTKs) has been implicated in drug resistance in many cancer types^21–24^. The ALK^25^ plays a critical role in nervous system development during embryogenesis. In contrast, its expression and activity in normal adult human tissue are limited^26,27^. However, increased ALK activity through protein overexpression, gene amplification, gene rearrangements and/or gain-of-function mutations has been implicated in the pathogenesis of many cancers^28–31^. Several selective ALK inhibitors have been FDA approved for use in patients whose tumors display evidence of increased ALK activity^32^. The selective nature of such ALK inhibitors is associated with greater tolerability due to limited off-target effects^33^. ALK positivity, as assessed by immunohistochemistry (IHC), is present in 20.9% and 36% of ovarian and breast cancers, respectively, and more prevalent in aggressive histological subtypes, such as high-grade serous ovarian cancer (HGSOC) (28% in HGSCO versus 0% in other ovarian cancer subtypes, *P* = 0.002) and triple-negative breast cancer (TNBC) (47% in TNBC versus 34% in non-TNBC, *P* = 0.0034)^30,31^. Furthermore, ALK overexpression is associated with shorter recurrence-free survival in patients with breast cancer^30^. These findings suggest that ALK plays a critical role in driving the malignant phenotype in ovarian and breast cancers. In the present study, we demonstrate that phosphorylation of ALK is higher in PARP inhibitor-resistant ovarian and breast cancers compared with their PARP inhibitor-sensitive counterparts and present evidence implicating the ALK–CDK9 axis as a driver of PARP inhibitor resistance which can be effectively overcome by selective ALK inhibitors. The direct regulation of CDK9 by p-ALK demonstrated here uncovers a critical mechanistic link of how membrane receptors modulate the DNA-damage response pathway via RNA polymerase (Pol) II-dependent transcriptional activation. Collectively, our findings provide a biomarker-driven, combinatorial treatment strategy to overcome PARP inhibitor resistance.

## Results

### ALK inhibition overcomes resistance to PARP inhibitors

**Fig. 1:**
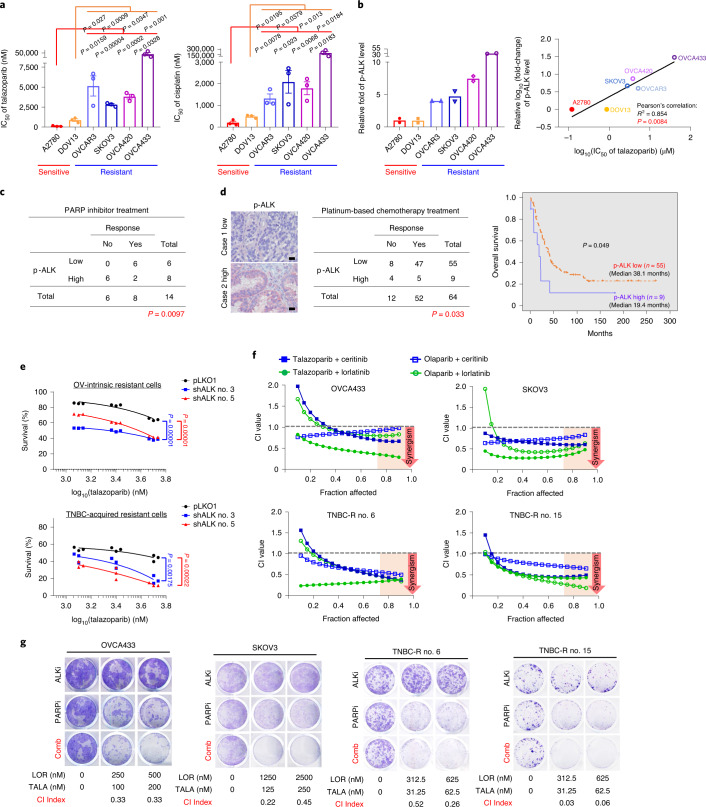
ALK inhibition demonstrates synergistic effects with PARP inhibitors in vitro. **a**, IC_50_ of the PARP inhibitor, talazoparib (left panel) and cisplatin (right panel). Ovarian cancer cells were treated with talazoparib or cisplatin for 6 d and subjected to MTT assay to determine cell viability. Error bars represent mean ± s.e.m. of *n* = 3 independent experiments, two-tailed, unpaired Student’s *t*-test. **b**, Left: quantification of phosphorylation signals of ALK in PARP inhibitor-sensitive and PARP inhibitor-resistant ovarian cancer cells. Cell lysates were analyzed using the Human Phospho-RTK Array Kit following the manufacturer’s instructions. Quantified phosphorylation signal was derived from two antibody spots of ALK. Right: correlations between IC_50_ of PARP inhibitor (talazoparib) and phosphorylation signals of ALK in PARP inhibitor-/platinum-sensitive and PARP inhibitor-/platinum-resistant ovarian cancer cells (Pearson’s correlation coefficient; *P* = 0.0084). Independent experiments (*n* = 3) of MTT assay for calculating the IC_50_ of PARP inhibitors. **c**, Correlation between clinical responses to PARP inhibitor (olaparib) and expression of p-ALK (high p-ALK (*n* = 8 patients) versus low p-ALK (*n* = 6 patients) in patients with ovarian cancer (two-sided Fisher’s exact test; *P* = 0.0097). **d**, Left: representative images and correlation between clinical responses to cisplatin/carboplatin and expression of p-ALK (high p-ALK (*n* = 9 patients) versus low p-ALK (*n* = 55 patients) in patients with ovarian cancer (two-sided Fisher’s exact test; *P* = 0.033). Scale bar, 20 µm. Right: Kaplan–Meier overall survival curves of patients with ovarian cancer, stratified by p-ALK expression levels (high p-ALK (*n* = 9 patients) versus low p-ALK (*n* = 55 patients; *P* = 0.049). **e**, Cell viability of ALK-knockdown PARP inhibitor-resistant cells treated with the indicated concentration of talazoparib for 6 d. Cell survival is calculated as the percentage relative to the control treatment in each group. OV, ovarian. Data represent *n* = 3 independent experiments, two-tailed, unpaired Student’s *t*-test. **f**, Chou–Talalay analysis of PARP inhibitor-resistant ovarian or TNBC cells treated with varying concentrations of PARP inhibitors (talazoparib or olaparib) and ALK inhibitors (ceritinib or lorlatinib) for 6 d. Synergism showed as CI < 1 at an optimal effect level (Fa > 0.75). The mean percentage of growth inhibition derived from *n* = 3 independent experiments of the MTT assay was used to calculate the CI value. **g**, Representative images of clonogenic assay results in PARP inhibitor (PARPi)-resistant ovarian and TNBC cells in the presence of the indicated inhibitor for 12 d. ALKi, ALK inhibitor; LOR, lorlatinib; TALA, talazoparib; Comb, combination of lorlatinib and talazoparib. The mean percentage of growth inhibition derived from *n* = 3 independent experiments of the clonogenic assay was used to calculate the CI value. Synergistic inhibition of cell proliferation is defined as CI < 1. Source data

**Extended Data Fig. 1:**
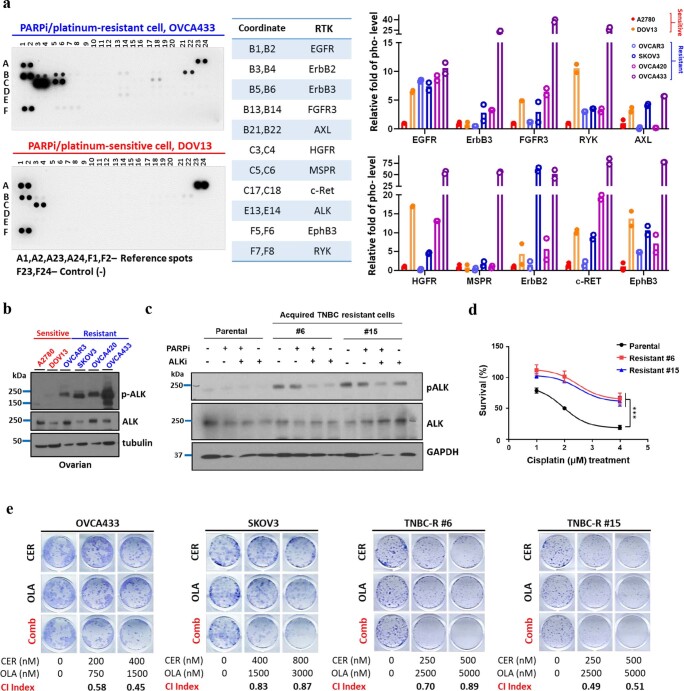
Phosphorylation of ALK is increased in PARPi/platinum-resistant ovarian and breast cancer. (**a**) Representative images (left) and quantification of phosphorylation signals (right) of receptor tyrosine kinases (RTKs) in PARPi-sensitive and PARPi-resistant ovarian cancer cells. Cell lysates were analyzed using the Human Phospho-RTK Array Kit (ARY001B, R&D Systems) following the manufacturer’s instructions. The quantification for p-ALK is included in the main manuscript (Fig. 1B, left panel). Quantified phosphorylation signals derived from n = 2 antibody spots of indicated RTK. (**b**) Western blot analysis of indicated proteins in PARPi/platinum-sensitive and PARPi/platinum-resistant ovarian cancer cells. Data are representative of two repeats with similar results. (**c**) Western blot analysis of indicated proteins in PARPi-sensitive triple-negative breast cancer (TNBC) cells (parental) and TNBC cells with acquired resistance to PARPi (#6 and #15) treated with 50 nM PARPi (talazoparib) or 0.5 μM ALK inhibitor (ALKi; ceritinib), either alone or in combination, for 48 hours. TNBC cells with acquired resistance to PARPi were selected after exposing PARPi-sensitive SUM149 cells to the increasing doses of talazoparib. Data are representative of two repeats with similar results. (**d**) Cell viability of parental PARPi-sensitive TNBC cells or TNBC cells with acquired resistance to PARPi treated with the indicated dose of cisplatin for 96 hours. Data are represented as mean ± SD of N = 3 independent experiments. Parental vs Resistant #6, ***P = 0.0004; Parental vs Resistant #15, ***P = 0.0006; one-way ANOVA analysis. (**e**) Representative images of clonogenic assay results in PARPi-resistant ovarian and TNBC cells in the presence of the indicated inhibitor for 12 days. The mean percentage of growth inhibition derived from N = 3 independent experiments of clonogenic assay was used to calculate the combination index (CI) value. Synergistic inhibition of cell proliferation is defined as CI < 1. CER, ceritinib; OLA, olaparib; Comb, combination of ceritinib and olaparib. Source data

Several PARP inhibitors have demonstrated efficacy in the treatment of platinum-sensitive ovarian cancers, suggesting that response to PARP inhibitors is closely related to sensitivity to platinum-based chemotherapy^34^. As resistance to platinum-based chemotherapy is a strong predictor of PARP inhibitor resistance^35^, we examined the cytotoxic effects of a PARP inhibitor (talazoparib) and a platinum compound (cisplatin) in *BRCA*–wild-type (WT) ovarian cancer cells. Indeed, the sensitivity of cells to PARP inhibitors closely mirrored that of the platinum compound (Fig. 1a). On the basis of the above results, we divided those cells into two groups: PARP inhibitor/platinum sensitive and PARP inhibitor/platinum resistant (Fig. 1a). To identify biomarkers predicting PARP inhibitor resistance that can simultaneously serve as actionable targets, we performed a human phospho-RTK (p-RTK) antibody array analysis using lysates obtained from those cells. We first sought to identify p-RTKs that were consistently expressed at higher levels in PARP inhibitor-/platinum-resistant cells compared with PARP inhibitor-/platinum-sensitive cells. It is interesting that quantification of p-RTK expression as assessed by the antibody array demonstrated that p-ALK expression was consistently higher in PARP inhibitor-/platinum-resistant cell lines compared with PARP inhibitor-/platinum-sensitive cell lines (Fig. 1b, left panel, and Extended Data Fig. 1a). In addition, p-ALK expression was significantly correlated with PARP inhibitor sensitivity in these cell lines (Fig. 1b, right panel). Western blot (WB) analysis recapitulated results from the p-RTK antibody array and detected higher p-ALK expression in PARP inhibitor-/platinum-resistant cells (Extended Data Fig. 1b). In human ovarian cancer tissue samples, expression of p-ALK was associated with resistance to PARP inhibitors (Fig. 1c) and platinum-based (cisplatin/carboplatin) chemotherapy (Fig. 1d, left panel). Moreover, p-ALK expression was also associated with shorter overall survival in patients with ovarian cancer treated with platinum-based chemotherapy (Fig. 1d, right panel). In support of the role of ALK in acquired resistance to PARP inhibitors, we also observed increased p-ALK expression in TNBC cells with acquired resistance to PARP inhibitors (nos. 6 and 15) compared with PARP inhibitor-sensitive TNBC cells (parental; Extended Data Fig. 1c). In line with the results in PARP inhibitor-/platinum-resistant ovarian cancer cells, TNBC cells with acquired resistance to PARP inhibitors also showed increased resistance to cisplatin compared with PARP inhibitor-sensitive parental cells (Extended Data Fig. 1d). Together, these data suggest that p-ALK is associated with resistance to PARP inhibitor/platinum therapy in ovarian and breast cancer. To further investigate the role of ALK in PARP inhibitor resistance, we depleted ALK in PARP inhibitor-resistant cells and found that depletion of ALK re-sensitized PARP inhibitor-resistant cells to talazoparib (Fig. 1e). In addition, we observed synergistic suppression of cell growth in vitro in both intrinsic and acquired resistant cells as indicated by a combination index (CI) value of <1 when cells were treated with PARP inhibitors and ALK inhibitors across a wide range of molar ratios (Fig. 1f). Similarly, combining PARP and ALK inhibitors resulted in strong synergistic inhibition of colony formation (Fig. 1g and Extended Data Fig. 1e). Collectively, these results indicated that ALK plays an important role in intrinsic and acquired resistance to PARP inhibitors.

### ALK inhibition blocks HR repair in PARP inhibitor-resistant cells

**Fig. 2:**
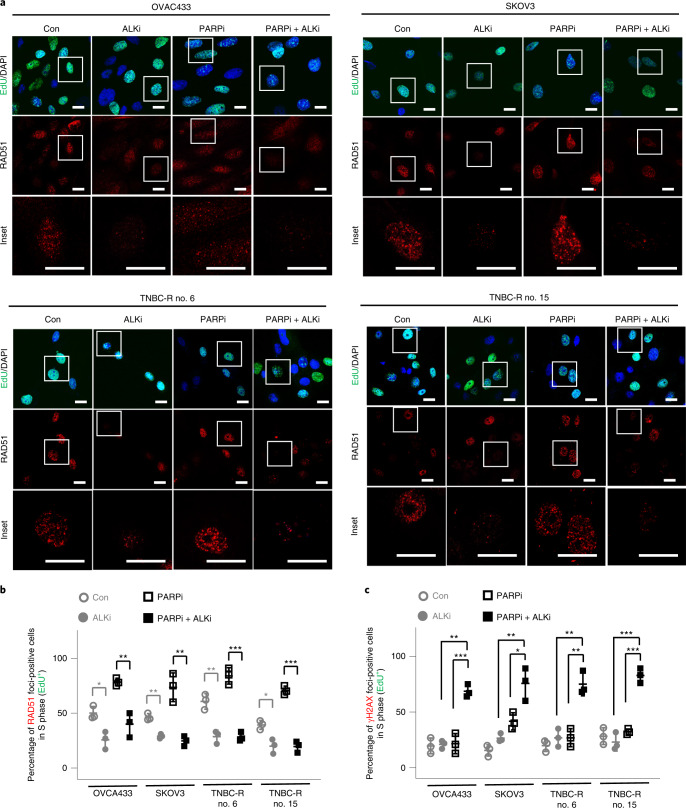
ALK inhibition reduces HR-repair activity in response to DNA damage. **a**, Representative images of RAD51 with EdU/DAPI staining in PARP inhibitor-resistant ovarian and TNBC cells treated with 0.25 μM PARP inhibitor (PARPi; talazoparib) or 0.5 μM ALK inhibitor (ALKi; lorlatinib), either alone or in combination, for 48 h. Insets: ×3.3 magnification. Scale bar, 20 µm. Data represent *n* = 3 independent experiments with similar results. **b**,**c**, Quantification of EdU-positive cells with RAD51 (**b**) and γH2AX (**c**) foci in PARP inhibitor-resistant ovarian and TNBC cells treated with 0.25 μM PARP inhibitor (talazoparib) or 0.5 μM ALK inhibitor (lorlatinib), either alone or in combination, for 48 h. Error bars represent mean ± s.d. of *n* = 3 independent experiments. Statistical analysis was carried out using the two-tailed, unpaired Student’s *t*-test **b**, Control (Con,○) versus ALK inhibitor (ALKi, ●): ^*^P = 0.0141 in OVCA433, ^**^P = 0.0014 in SKOV3, ^**^P = 0.0038 in TNBC-R no. 6, ^*^P = 0.0122 in TNBC-R no. 15; PARP inhibitor (PARPi, □) versus PARP inhibitor + ALK inhibitor (PARPi + ALKi, ■): ^**^P = 0.0047 in OVCA433, ^**^P = 0.0036 in SKOV3, ^***^P = 0.0004 in TNBC-R no. 6, ^***^P = 0.0002 in TNBC-R no. 15. **c**, PARP inhibitor (PAPRi, □) versus PARP inhibitor + ALK inhibitor (PARPi + ALKi, ■): ^***^P = 0.0003 in OVCA433, ^*^P = 0.0235 in SKOV3, ^**^P = 0.0032 in TNBC-R no. 6, ^***^P = 0.0002 in TNBC-R no. 15; ALK inhibitor (ALKi, ●) versus PARP inhibitor + ALK inhibitor (PARPi + ALKi, ■): ^**^P = 0.0016 in OVCA433, ^**^P = 0.005 in SKOV3, ^**^P = 0.0032 in TNBC-R no. 6, ^***^P = 0.0005 in TNBC-R no. 15. Source data

**Extended Data Fig. 2:**
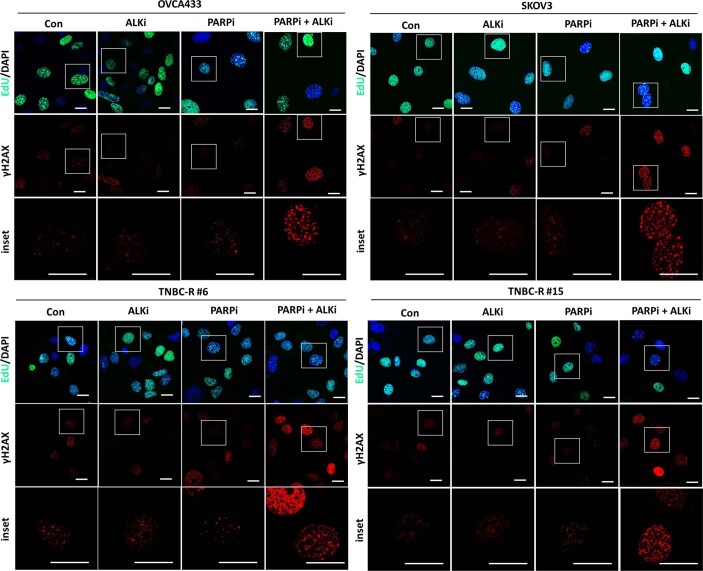
Combined inhibition of PARP and ALK results in accumulation of DNA damage. Representative images of γH2AX with EdU/DAPI staining in PARP inhibitor (PARPi)-resistant ovarian and triple-negative breast cancer (TNBC) cells treated with 0.25 μM PARPi (talazoparib) or 0.5 μM ALK inhibitor (ALKi; lorlatinib), either alone or in combination, for 48 hours. Insets, 3.3× magnification. Bar, 20 µm. Data are representative of N = 3 independent experiments with similar results.

**Extended Data Fig. 3:**
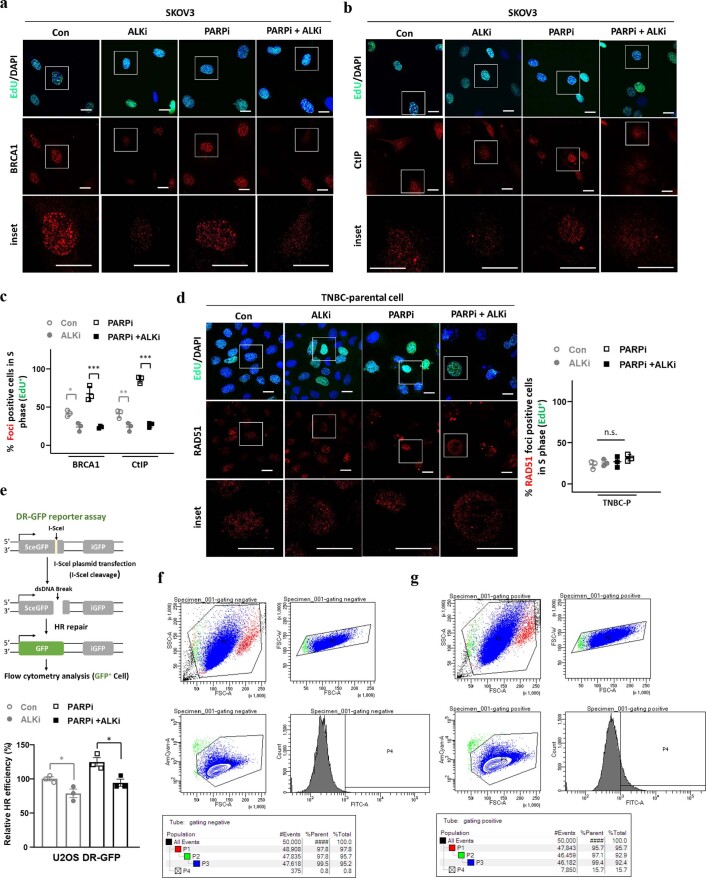
ALK inhibition suppresses the HR efficiency. (**a, b**) Representative images of BRCA1 (**a**) and CtIP (**b**) with EdU/DAPI staining in PARP inhibitor (PARPi)-resistant ovarian cancer cells treated with 0.25 μM PARPi (talazoparib) or 0.5 μM ALK inhibitor (ALKi; lorlatinib), either alone or in combination, for 48 hours. Insets, 3.3× magnification. Bar, 20 µm. Data are representative of N = 3 independent experiments with similar results. (**c**) Quantification of EdU-positive cells with BRCA1 or CtIP foci in PARPi-resistant ovarian cancer cells treated with 0.25 μM PARPi (talazoparib) or 0.5 μM ALKi (lorlatinib), either alone or in combination, for 48 hours. Error bars represent mean ± SD of N = 3 independent experiments, one-way ANOVA analysis, n.s., not significant, Control (Con,○) vs ALK inhibitor (ALKi,●): *P = 0.0235 in BRCA1, ***P < 0.0001 in CtIP; PARP inhibitor + ALK inhibitor (PARPi+ALKi, ■) vs PARP inhibitor (PARPi, □): **P = 0.0091 in BRCA1, ***P < 0.0001 in CtIP. (**d**) Representative images (left) and quantification (right) of EdU positive cells with RAD51 foci in PARPi-sensitive (TNBC) treated with 0.25 μM PARPi (talazoparib) or 0.5 μM ALKi (lorlatinib), either alone or in combination, for 48 h. Insets, 3.3× magnification. Bar, 20 µm. Error bars represent mean ± SD of N = 3 independent experiments, one-way ANOVA analysis, n.s., not significant. (**e**) Top: Schematic of the DR-GFP reporter assay. Functional HR can repair DNA double-strand breaks and results in the expression of GFP. Bottom: HR efficiency in U2OS DR-GFP cells treated with 0.25 μM PARPi (talazoparib) or 0.5 μM ALKi (lorlatinib), either alone or in combination, for 48 hours. Flow cytometry analysis was used to measure the percentage of GFP + cells. Error bars represent mean ± SEM of N = 3 independent experiments, two-tailed unpaired t test, Control (Con,○) vs ALK inhibitor (ALKi, ■): *P = 0.0337; PARP inhibitor + ALK inhibitor (PARPi+ALKi, ■) vs PARP inhibitor (PARPi, □): *P = 0.0215. (**f, g**) Gating strategy to determine the percentage of GFP-positive cells in U2OS DR-GFP cells under (f) basal conditions or (g) after transfection with I-SceI expression plasmid. Source data

HR-deficient ovarian cancers are sensitive to both PARP inhibitors and platinum compounds^36^. In a recent clinical trial, the therapeutic agent that induces HR deficiency demonstrated efficacy to overcome resistance to PARP inhibitors^37^. Notably, our data (Fig. 1) indicated that p-ALK expression is associated with resistance to PARP inhibitors and platinum compounds. Thus, we sought to determine whether p-ALK increases HR activity. We assessed the foci formation of HR factors, including RAD51 (Fig. 2a), BRCA1 and C-terminal-binding protein and interacting protein (CtIP; Extended Data Fig. 3a,b) in PARP inhibitor-resistant cells by immunofluorescence (IF). In addition, we simultaneously assessed expression of 5-ethynyl-2′-deoxyuridine (EdU), an S-phase marker of the cell cycle (Fig. 2a and Extended Data Figs. 2 and 3a,b) and γH2AX for DNA damage (Extended Data Fig. 2). It is interesting that quantification of these IF results demonstrated that ALK inhibitor monotherapy reduced the percentage of PARP inhibitor-resistant cells with foci of HR factors in the S phase, suggesting that ALK inhibitor monotherapy results in a decrease in HR-repair activity compared with the control (**○**) group (Fig. 2b and Extended Data Fig. 3c). Next, we asked whether ALK inhibition induces an HR-deficient phenotype and consequent accumulation of DNA damage when used in combination with PARP inhibitors. Indeed, the combination of ALK and PARP inhibitors, compared with PARP inhibitors alone, abrogated foci formation of HR factors (■ PARPi + ALKi versus □ PARPi, Fig. 2b and Extended Data Fig. 3c) and increased γH2AX foci formation (■ PARPi + ALKi versus □ PARPi, Fig. 2c) in PARP inhibitor-resistant cells in the S phase. In addition, we sought to determine whether treatment with ALK inhibitor reduces HR activity in the PARP inhibitor-sensitive cells and found that RAD51 foci formation remains unchanged when treated by ALK inhibitor alone or in combination with PARP inhibitor (■, PARPi + ALKi, Extended Data Fig. 3d). These results suggest that the effects of ALK inhibitor on HR activity is specific to PARP inhibitor-resistant cells. For orthogonal validation of our findings, we performed an HR reporter assay under similar treatment conditions and found that pharmacological inhibition of ALK, either as monotherapy or in combination with PARP inhibition, resulted in reduced HR activity at the cellular level (Extended Data Fig. 3e). Collectively, these data demonstrate that ALK inhibition in PARP inhibitor-resistant cells suppresses HR activity which, when combined with PARP inhibition, results in accumulation of DNA damage.

**Extended Data Fig. 4:**
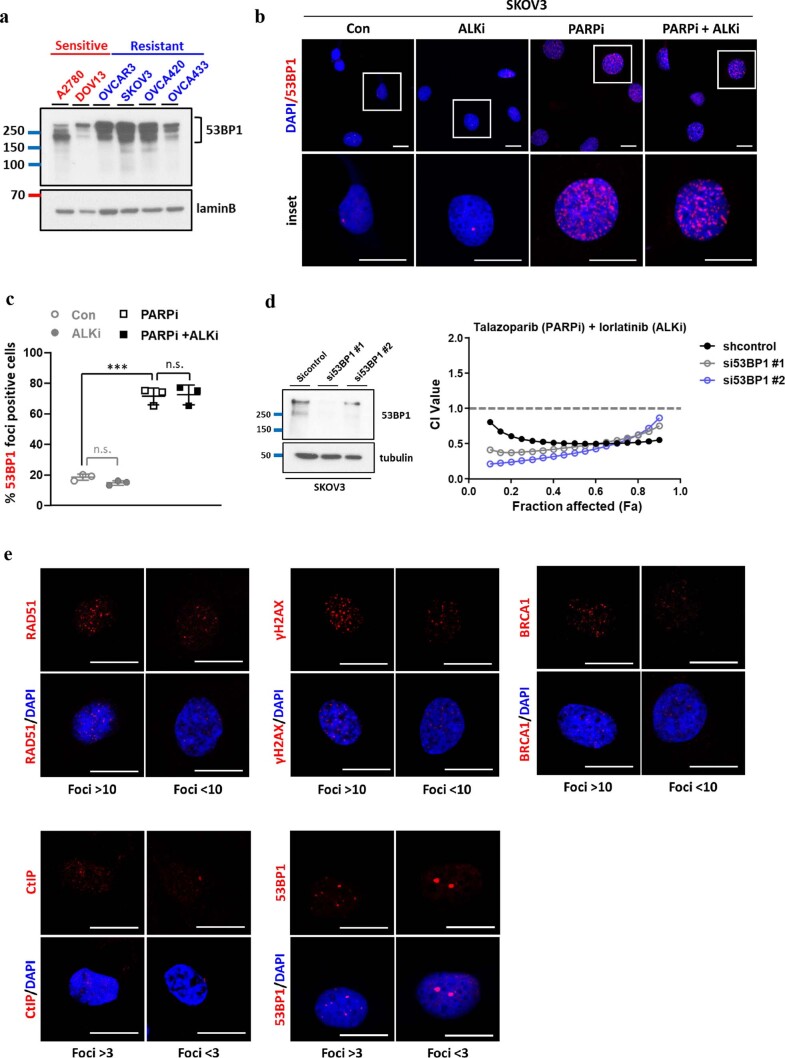
Relationship between 53BP1-dependent NHEJ and ALKi-mediated sensitization to the PARP inhibition. (**a**) Western blot analysis of 53BP1 expression in PARPi-sensitive and PARPi-resistant ovarian cancer cells. Data are representative of two repeats with similar results. (**b, c**) Representative images of 53BP1 with DAPI staining (**b**) and quantification of 53BP1 foci–positive cells (**c**) in PARPi-resistant ovarian cancer cells treated with 0.25 μM PARPi (talazoparib) or 0.5 μM ALKi (lorlatinib), either alone or in combination, for 48 hours. Error bars represent mean ± SD of N = 3 independent experiments. Insets, 3× magnification. Bar, 20 µm. n.s., not significant, Control (Con, ○) vs PARP inhibitor (PARPi,■): ***P = 0.00009, two-tailed unpaired t test. (**d**) PARPi-resistant SKOV3 cells were transfected with control siRNA (siControl or siRNAs targeting 53BP1 (si53BP1#1, si53BP1#2)). Relative expression levels of total 53BP1 in cells were determined by Western blot analysis (left panel). Chou-Talalay analysis of PARPi-resistant, knockdown control, or 53BP1-depleted cells treated with varying concentrations of PARPi (talazoparib) and ALKi (lorlatinib) for 6 days (right panel). The mean percentage of growth inhibition derived from N = 3 independent MTT experiments was used to calculate the combination index (CI) value. Synergistic inhibition of cell proliferation is defined as CI < 1. (**e**) Representative images of cells that are just above and below positivity thresholds for each marker as indicated. Bar, 20 µm. Data are representative of N = 3 independent experiments with similar results. Source data

As the synthetically lethal interaction between HR deficiency and PARP inhibition has been linked to upregulation of toxic 53BP1-dependent nonhomologous end-joining (NHEJ) activity, we next sought to determine whether ALK inhibitor-mediated sensitization to PARP inhibition is dependent on 53BP1-mediated NHEJ in the setting of HR deficiency. We first examined 53BP1 expression in PARP inhibitor-sensitive and PARP inhibitor-resistant cells, and found that 53BP1 was not reduced in PARP inhibitor-resistant cells compared with PARP inhibitor-sensitive cells (Extended Data Fig. 4a). As recruitment of 53BP1 at the DNA damage site is important to promote the NHEJ activity, we next assessed 53BP1 foci formation under various treatment conditions and found that ALK inhibitor monotherapy did not significantly increase 53BP1 foci formation in PARP inhibitor-resistant cells (● ALKi versus ○ Con, in Extended Data Fig. 4b,c). In contrast, PARP inhibitor monotherapy (□) significantly increased 53BP1 foci formation in PARP inhibitor-resistant cells compared with the control (○) group (in Extended Data Fig. 4b,c). Notably, combination of ALK and PARP inhibitors did not further increase 53BP1 foci formation compared with PARP inhibitors alone (■ PARP inhibitor + ALK inhibitor versus □ PARP inhibitor, in Extended Data Fig. 4b,c). These results suggest that ALK inhibitor is unable to increase 53BP1-mediated NHEJ activity. Notably, depletion of 53BP1 did not compromise the observed synergy between ALK and PARP inhibitors in PARP inhibitor-resistant cells (Extended Data Fig. 4d). Collectively, these results suggest that 53BP1-dependent, toxic end-joining is not a major mechanism of ALK inhibitor-mediated sensitization to PARP inhibition.

### ALK promotes PARP inhibitor resistance via phosphorylating Tyr19-CDK9

**Fig. 3:**
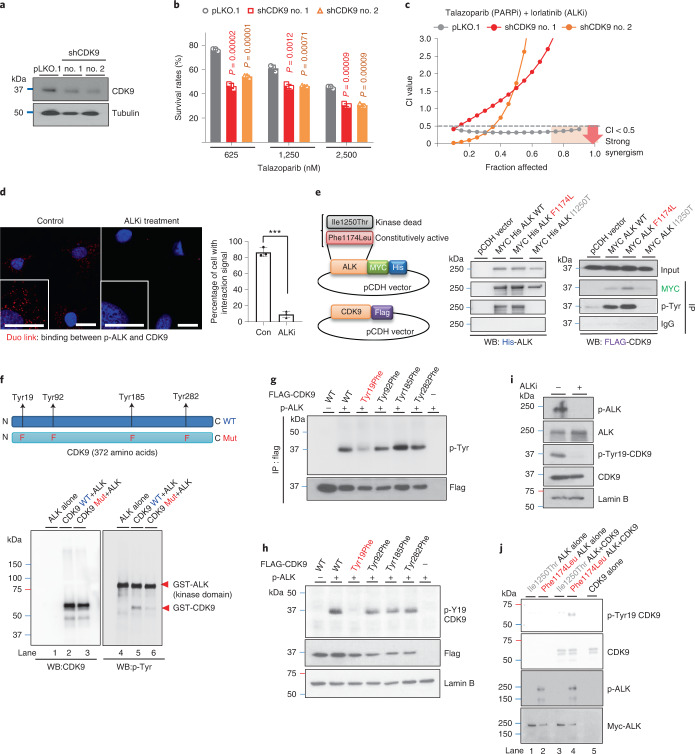
ALK directly phosphorylates CDK9 at Tyr19. **a**, PARP inhibitor-resistant SKOV3 cells were infected with control short hairpin (sh)RNA (pLKO.1) or shRNAs targeting CDK9 (shCDK9 no. 1/ shCDK9 no. 2). CDK9 expression in stable clones was determined by WB. Data represent two repeats with similar results. **b**, Cell viability of CDK9-knockdown PARP inhibitor-resistant cells treated with the indicated concentration of talazoparib for 6 d. Error bar represents mean ± s.d. of *n* = 3 independent experiments, two-tailed, unpaired Student’s *t*-test. **c**, Chou–Talalay analysis of CDK9-knockdown (shCDK9 no. 1/shCDK9 no. 2) and control-knockdown (pLKO.1) PARP inhibitor-resistant cells treated with varying concentrations of talazoparib and CDK9 inhibitor for 6 d. The mean percentage of growth inhibition derived from *n* = 3 independent MTT experiments was used to calculate the CI value. Strong synergism showed as CI < 0.5 at an optimal effect level (Fa > 0.75, region highlighted in orange). The CI values at Fa > 0.75: pLKO.1 < 0.5 < shCDK9 no. 1 < shCDK9 no. 2. **d**, PARP inhibitor-resistant SKOV3 cells treated with or without 0.5 μM ALK inhibitor (lorlatinib) for 24 h. Detection of p-ALK and CDK9 binding (red dots) was performed by Duo-link assay. Insets: ×2 magnification. Scale bar, 20 µm. Bar diagram shows the percentage of cells with positive interaction calculated from *n* = 3 independent experiments. Error bar represents mean ± s.d. ^***^P = 0.00003, two-tailed, unpaired Student’s *t*-test. **e**, Left: schematics of CDK9 and different ALK-expressing plasmids. Right: WB of FLAG-tagged CDK9 in cells coexpressing WT, constitutively active (Phe1174Leu) or kinase-dead (Ile1250Thr) ALK with FLAG-tagged CDK9 after IP with the indicated antibodies. Data represent two repeats with similar results. **f**, WB of tyrosine phosphorylation (p-Tyr) signal and CDK9 in an in vitro kinase assay of purified ALK incubated with WT or Tyr/Phe-mutant (phenylalanine mutation of Tyr19/Tyr92/Tyr185/Tyr282) CDK9 protein. Data represent two repeats with similar results. **g**,**h**, Cells expressing exogenous WT, Tyr19Phe, Tyr92Phe, Tyr185Phe or Tyr282Phe CDK9 with or without constitutively active ALK. The p-Tyr signal and FLAG-tagged CDK9 were examined by WB after IP with FLAG antibody (**g**). The p-Tyr19-CDK9 expression of FLAG-tagged CDK9 was examined by WB (**h**). **i**, WB of indicated proteins in cells treated with or without 0.5 μM ALK inhibitor (lorlatinib) for 24 h. **j**, WB of in vitro kinase assay in which purified GST–CDK9 was incubated with constitutively active (Phe1174Leu) or kinase-dead ALK (Ile1250Thr) proteins. Data represent two repeats with similar results. Source data

**Extended Data Fig. 5:**
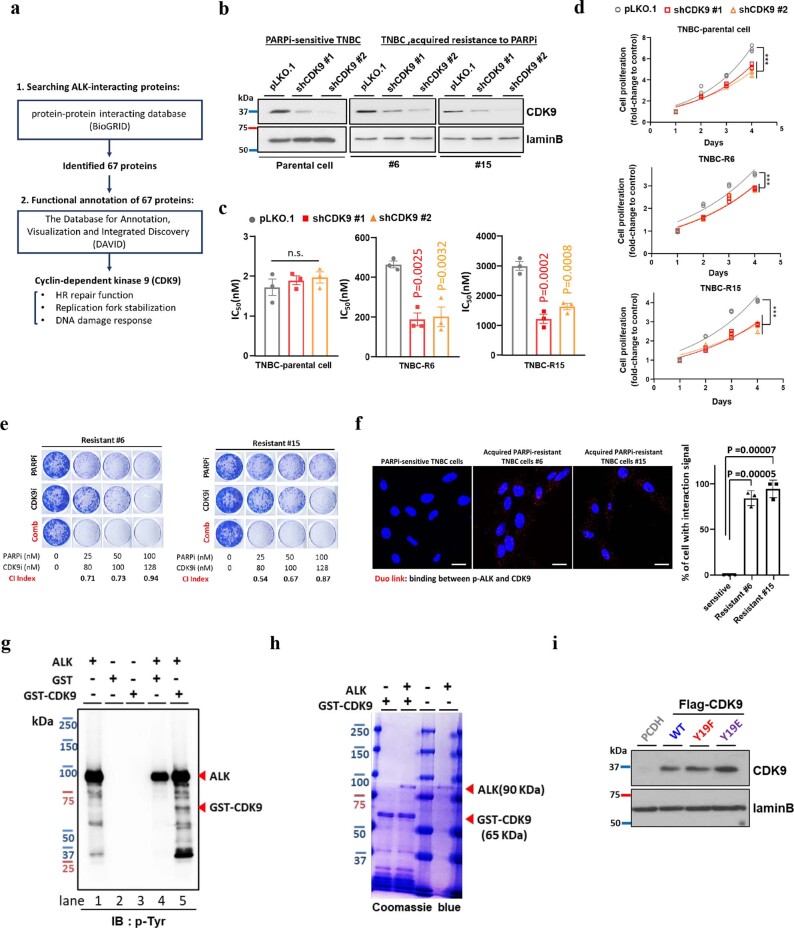
ALK tyrosine phosphorylates CDK9 at Y19 to promote resistance to PARP inhibitor. (**a**) Schematic of the strategy using the indicated criteria (1 and 2) to identify proteins with DNA repair function that potentially regulated by ALK. (**b**) Cells were infected with control shRNA (pLKO.1) or shRNAs targeting the CDK9 (shCDK9 #1/ shCDK9#2). Relative expression levels of total CDK9 in stable clones were determined by WB. Data are representative of two repeats with similar results. (**c**) Half maximal inhibitory concentration (IC50) of PARPi (talazoparib). CDK9-knockdown, PARPi-resistant, and PARPi-sensitive cells were treated with talazoparib for 6 days and subjected to MTT assay to determine cell viability. Error bars represent mean ± SEM of N = 3 independent experiments. (**d**) Cell growth of CDK9-knockdown PARPi-resistant and PARPi-sensitive cells on indicated days. Data represent three independent experiments. (**e**) Representative images of clonogenic assay in TNBC cells with acquired resistance to PARPi in the presence of the indicated inhibitor for 12 days. The mean percentage survival derived from N = 3 independent experiments was used to calculate the combination index (CI) value. Synergistic inhibition of cell proliferation is defined as CI < 1. (**f**) Detection of p-ALK and CDK9 binding (red dots) in PARPi-sensitive TNBC parental cells and TNBC cells with acquired resistance to PARPi (#6 and #15), determined by Duo-link assay. Bar, 20 µm. Bar diagram, the percentage of cells showing positive interaction calculated. Error bar represent mean ± SD of N = 3 independent experiments. (**g**) WB of tyrosine phosphorylation (p-Tyr) signal in in vitro kinase assay results in which purified ALK was incubated with GST-CDK9 protein. Data are representative of two repeats with similar results. (**h**) Coomassie blue staining of purified ALK and GST-CDK9 protein. Data are representative of two repeats with similar results. (**i**) Stable re-constitution of PCDH (vector control), wild type (WT), Y19E, or Y19F CDK9 in SKOV3 cells depleted of endogenous CDK9. Expression of Flag-tagged CDK9 was determined by Western blot analysis. Data are representative of two repeats with similar results. Statistical analysis was carried out using the one-way ANOVA analysis (**c, d**) and two-tailed unpaired t test (**f**). (**c**) n.s., not significant. (**d**) pLKO.1 vs shCDK9#1: ***P = 0.0003 in Parental, ***P < 0.0001 in R#6 and R#15; pLKO.1 vs shCDK9#2: ***P < 0.0001 in Parental, R#6 and R#15. Source data

To elucidate the detailed mechanisms of ALK-mediated PARP inhibitor resistance, we searched for ALK-interacting proteins with DNA damage and/or repair functions. Utilizing the online protein–protein interacting database, BioGRID, we identified 67 proteins known to interact with ALK. Notably, functional annotation of these 67 proteins using the Database for Annotation, Visualization and Integrated Discovery (DAVID) identified cyclin-dependent kinase 9 (CDK9), a transcriptionally associated CDK known to play important roles in regulating HR function, replication fork stabilization and DNA-damage response^38,39^ (Extended Data Fig. 5a). To further determine whether CDK9 activity is important for ALK-mediated PARP inhibitor resistance, we depleted CDK9 in PARP inhibitor-resistant cells with high expression of p-ALK and demonstrated that depletion of CDK9 re-sensitized PARP inhibitor-resistant cells to PARP inhibitors (Fig. 3a,b and Extended Data Fig. 5b,c). As CDK9 has been reported as a regulator of transcription and cancer cell growth, we further examined the proliferation rate of PARP inhibitor-resistant and inhibitor-sensitive cells depleted of CDK9. We found that depletion of CDK9 reduced the proliferation rate in both PARP inhibitor-resistant and inhibitor-sensitive cells (Extended Data Fig. 5d). However, in contrast to PARP inhibitor-resistant cells, half-maximal inhibitory concentration (IC_50_) values of PARP inhibitor in PARP inhibitor-sensitive cells remained unaffected by depletion of CDK9 (Extended Data Fig. 5c), suggesting that general reduction in cell growth alone is not sufficient to induce changes in the sensitivity to PARP inhibitors. These findings are further supported by results showing that the combination of PARP inhibitor and a CDK9 inhibitor resulted in synergistic inhibition of colony formation in PARP inhibitor-resistant cells (Extended Data Fig. 5e). Moreover, strong synergistic growth inhibition after treatment with the combination of ALK and PARP inhibitors was observed in knockdown control cells but not in CDK9-depleted cells (Fig. 3c), suggesting that ALK contributes to PARP inhibitor resistance by regulating CDK9 activity.

To further investigate whether ALK interacts with and tyrosine phosphorylates CDK9, we performed the Duo-link assay in PARP inhibitor-resistant cells treated with or without ALK inhibitor (Fig. 3d) and in TNBC cells sensitive to or with acquired resistance to PARP inhibitors (Extended Data Fig. 5f). The results indicated that the binding signal of p-ALK and CDK9 (Duo: red) was present in PARP inhibitor-resistant cells and inhibited after treatment with ALK inhibitor (Fig. 3d). Moreover, the increased binding signal was observed in the TNBC cells with acquired PARP inhibitor resistance compared with PARP inhibitor-sensitive TNBC parental cells (Extended Data Fig. 5f). Consistently, ALK interactions with CDK9 and tyrosine phosphorylation of CDK9 were detected in cells expressing WT and constitutively active (Phe1174Leu), but not kinase-dead (Ile1250Thr), ALK (Fig. 3e). These results suggested that CDK9 forms a complex with activated ALK and is tyrosine phosphorylated by ALK. To validate CDK9 as a substrate of ALK, we performed an in vitro kinase assay by incubating ALK with purified GST–CDK9 recombinant protein (Fig. 3f and Extended Data Fig. 5g). The molecular mass of ALK (90 kDa) and GST–CDK9 (65 kDa) recombinant proteins was analyzed by Coomassie Blue staining (red arrows; Extended Data Fig. 5h). The tyrosine phosphorylation signal was detected in GST–CDK9 (65 kDa) when it was incubated with ALK (Fig. 3f and Extended Data Fig. 5g), suggesting that ALK directly tyrosine phosphorylates CDK9. Next, utilizing a kinase-specific phosphorylation site-prediction program, GPS 3.0, we identified four potential tyrosine residues (Tyr19, Tyr92, Tyr185 and Tyr282) on CDK9 predicted to be phosphorylated by ALK (Fig. 3f, upper panel). By means of an in vitro kinase assay, we observed decreased phosphorylation of purified Tyr/Phe-mutant CDK9 protein (Tyr19Phe/Tyr92Phe/Tyr185Phe/Tyr282Phe) compared with WT CDK9 when incubated with ALK (Fig. 3f). We next generated various CDK9 mutants with the single phenylalanine mutation of each potential tyrosine residues and found that only the Tyr19Phe mutation alone abrogated tyrosine phosphorylation of CDK9 (Fig. 3g). To further support the existence of CDK9 Tyr19 phosphorylation (p-Tyr19-CDK9), we generated a specific antibody against p-Tyr19-CDK9. Consistently, p-Tyr19 of WT CDK9 was detected but no phosphorylation signal was observed in the Tyr19Phe CDK9 mutant when coexpressed with constitutively active ALK (Fig. 3h), and treatment with ALK inhibitor abolished p-Tyr19-CDK9 (Fig. 3i). The above findings are further supported by results showing that the p-Tyr19 signal of CDK9 was detected only when GST–CDK9 was incubated with constitutively active (Phe1174Leu) ALK, but not when incubated with kinase-dead (Ile1250Thr) ALK (Fig. 3j). Together, these findings suggest ALK-mediated tyrosine phosphorylation of CDK9 at Tyr19.

**Fig. 4:**
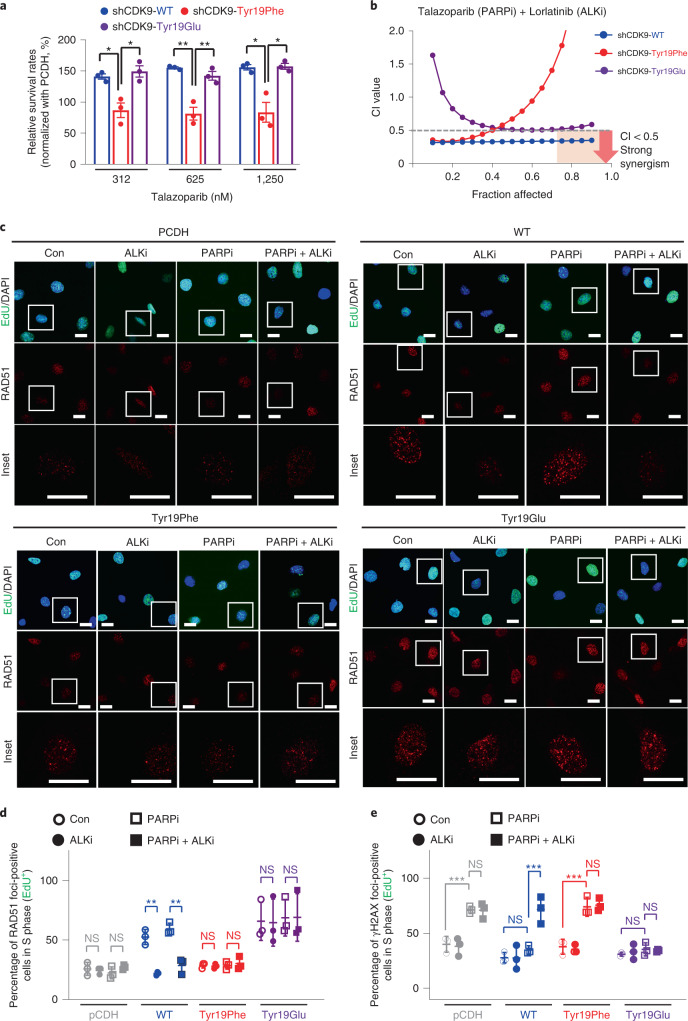
ALK promotes PARP inhibitor resistance and HR via phosphorylation of CDK9 at Tyr19. **a**, Relative cell viability of CDK9-knockdown SKOV3 cells re-expressing WT, Tyr19Glu or Tyr19Phe CDK9. Cells were treated with the indicated concentration of talazoparib for 6 d. Error bars represent mean ± s.d. of *n* = 3 independent experiments, two-tailed, unpaired Student’s *t*-test: Tyr19Phe versus WT at 312 nM: ^*^P = 0.012; Tyr19Phe versus Tyr19Glu at 312 nM: ^*^P = 0.013; Tyr19Phe versus WT at 625 nM: ^**^P = 0.002; Tyr19Phe versus Tyr19Glu at 625 nM: ^**^P = 0.008; Tyr19Phe versus WT at 1,250 nM: ^*^P = 0.0117; Tyr19Phe versus Tyr19Glu at 1,250 nM: ^*^P = 0.0111. **b**, Chou–Talalay analysis of CDK9-knockdown SKOV3 cells re-expressing WT, Tyr19Glu or Tyr19Phe CDK9. Cells were treated with varying concentrations of PARP inhibitor (talazoparib) and ALK inhibitor (lorlatinib) for 6 d. The mean percentage of growth inhibition derived from *n* = 3 independent MTT experiments was used to calculate the CI value. Strong synergism showed as CI < 0.5 at an optimal effect level (Fa > 0.75, region highlighted in orange). The CI values at Fa > 0.75: WT < 0.5 < Tyr9Glu < Tyr19Phe. **c**, Representative images of DAPI, RAD51 and EdU staining in PARP inhibitor-resistant SKOV3 cells depleted of endogenous CDK9 and re-constituted with pCDH (vector control), WT, Tyr19Glu or Tyr19Phe CDK9. These cells were cultured with 0.25 μM PARP inhibitor (talazoparib) or 0.5 μM ALK inhibitor (lorlatinib), either alone or in combination, for 48 h. Insets: ×3.3 magnification. Scale bar, 20 µm. Data represent *n* = 3 independent experiments with similar results. **d**,**e**, Quantification of EdU-positive cells with RAD51 foci (**d**) and γH2AX (**e**) foci in PARP inhibitor-resistant SKOV3 cells depleted of endogenous CDK9 and re-constituted with WT, Tyr19Glu or Tyr19Phe CDK9. These cells were cultured with 0.25 μM PARP inhibitor (talazoparib) or 0.5 μM ALK inhibitor (lorlatinib), either alone or in combination, for 48 h. Error bars represent mean ± s.d. of *n* = 3 independent experiments. Two-way ANOVA analysis: control versus ALK inhibitor in WT: ^**^P = 0.0027, PARP inhibitor versus PARP inhibitor + ALK inhibitor in WT: ^**^P = 0.0028 (**d**); control versus PARP inhibitor in PCDH: ^***^P < 0.001, control versus PARP inhibitor in Tyr19Phe: ^***^P < 0.001 (**e**). NS, not significant. Source data

**Extended Data Fig. 6:**
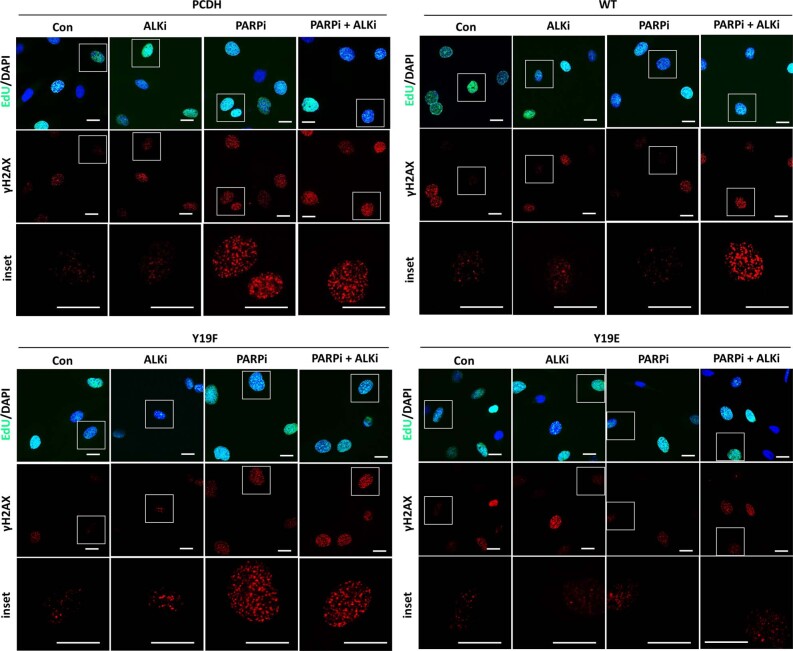
Inhibition of ALK-mediated phosphorylation of CDK9 at Y19 results in accumulation of DNA damage after treatment with PARPi in PARPi-resistant cells. Representative images of DAPI, γH2AX, and EdU staining in PARPi-resistant SKOV3 cells depleted of endogenous CDK9 and re-constituted with PCDH (vector control), WT, Y19E, or Y19F CDK9. These cells were cultured with 0.25 μM PARPi (talazoparib) or 0.5 μM ALKi (lorlatinib), either alone or in combination, for 48 hours. Insets, 3.3× magnification. Bar, 20 µm. Data are representative of N = 3 independent experiments with similar results.

To further investigate the functional importance of CDK9 Tyr19 phosphorylation in PARP inhibitor resistance, we generated stable cell lines re-expressing WT, Tyr19Phe (nonphosphomimetic) or Tyr19Glu (phosphomimetic) CDK9 in CDK9-depleted cells (Extended Data Fig. 5i). Notably, we found that re-expression of WT or Tyr19Glu CDK9, but not Tyr19Phe CDK9, restored a PARP inhibitor-resistant phenotype, as shown by increased relative cell survival rates normalized to vector control, (pCDH, empty vector without protein expression) (Fig. 4a). To further validate the role of p-Tyr19-CDK9 in ALK-mediated PARP inhibitor resistance and HR-repair activity, CDK9-depleted cells stably re-expressing WT, Tyr19Glu or Tyr19Phe CDK9 were treated with the combination of ALK and PARP inhibitors. It is interesting that we found that the strong synergy observed in cells re-expressing WT CDK9 is similar to that observed in the knockdown control cells shown in Fig. 3c. Compared with cells re-expressing WT CDK9, the CI values in cells re-expressing Tyr19Phe were higher at each fraction affected (Fa) point and were >1 at Fa points >0.65 (Fig. 4b, red dots), a pattern similar to that observed in CDK9-depleted cells as shown in Fig. 3c. These data showed that re-expression of Tyr19Phe-mutant CDK9 in CDK9-depleted cells does not alter the CI value profile. In contrast, re-expression of WT CDK9 in CDK9-depleted cells altered the CI value profile (Fig. 4b, red versus blue dots). We also found that CI values in cells re-expressing Tyr19Glu lost the strong synergistic growth inhibition effect compared with that observed in cells re-expressing WT CDK9, and the CI value profile was the opposite of the results from Tyr19Phe (Fig. 4b, purple versus red dots), reflecting the constitutively activated nature of Tyr19Glu. These results suggest that combined PARP and ALK inhibition achieved a strong synergistic inhibition of cellular proliferation in cells expressing WT CDK9, but not in cells expressing Tyr19Phe or Tyr19Glu-mutant CDK9. In addition, treatment with an ALK inhibitor reduced RAD51 foci formation in cells expressing WT CDK9, but not in cells expressing Tyr19Glu or Tyr19Phe CDK9 (Fig. 4c,d). Together, these results suggest that p-Tyr19-CDK9 is required for ALK-mediated PARP inhibitor resistance and HR-repair activity. Next, we further examined γH2AX foci formation in these cells. As shown in Fig. 4e and Extended Data Fig. 6, treatment with PARP inhibitor alone resulted in an increase of γH2AX foci formation in cells expressing Tyr19Phe CDK9 but not in cells expressing WT or Tyr19Glu CDK9. Although treatment with PARP inhibitors alone did not increase γH2AX foci formation in cells expressing WT CDK9, combined treatment with PARP and ALK inhibitors resulted in increased γH2AX foci formation in cells expressing WT CDK9 (Fig. 4e and Extended Data Fig. 6). Collectively, these results suggest that inhibition of ALK-mediated phosphorylation of Tyr19-CDK9 impairs HR and results in DNA damage after treatment with PARP inhibitors in PARP inhibitor-resistant cells.

### The p-Tyr19-CDK9 stabilizes P-TEFb and activates HR-repair genes

**Fig. 5:**
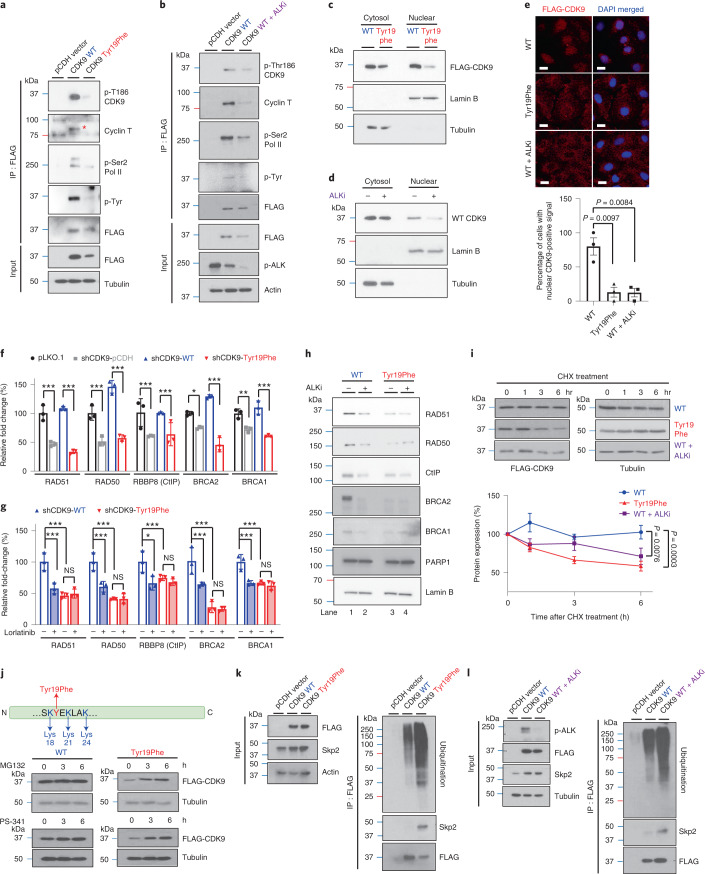
The p-Tyr19-CDK9 is important for its kinase activity and protein stability. **a**, Expression of the indicated proteins in SKOV3-stable cells expressing Tyr19Phe and WT CDK9 examined by WB after IP with FLAG antibody. Data represent two repeats with similar results. **b**, Expression of the indicated proteins in SKOV3-stable cells expressing WT CDK9 with or without ALK inhibitor treatment examined by WB after IP with FLAG antibody. Data represent two repeats with similar results. **c**,**d**, Subcellular localization of FLAG-tagged CDK9 in cells expressing Tyr19Phe (**c**) and WT CDK9 treated with or without ALK inhibitor (**d**). Data represent two repeats with similar results. **e**, Representative images of FLAG–CDK9 with DAPI staining (upper panel) and quantification of cells with nuclear CDK9-positive signal (lower panel) in cells expressing Tyr19Phe CDK9 and WT CDK9 treated with or without ALK inhibitors. Error bars represent mean ± s.e.m. of *n* = 3 independent experiments. Scale bar, 20 μM. **f**, Quantitative PCR analysis of gene expression in CDK9-knockdown SKOV3 cells rescued with WT or Tyr19Phe CDK9. Error bars represent mean ± s.d. of *n* = 3 independent experiments. **g**, Quantitative PCR analysis of gene expression in CDK9-knockdown SKOV3 cells rescued with WT or Tyr19Phe CDK9 treated with or without ALK inhibitors. Error bars represent mean ± s.d. of *n* = 3 independent experiments. **h**, WB of HR factors and PARP1 level in cells expressing WT or Tyr19Phe CDK9 after treatment with or without 0.5 μM ALK inhibitor (lorlatinib) for 24 h. Data represent two repeats with similar results. **i**, WB of FLAG-tagged CDK9 in SKOV3-stable cells expressing Tyr19Phe CDK9 and WT CDK9 treated with or without ALK inhibitors. Cells were treated with 50 μΜ CHX for the indicated time (upper panel). Quantification of band intensity is shown in the lower panel. Error bars represent mean ± s.e.m. of *n* = 3 independent experiments. **j**, WB of FLAG-tagged CDK9 in SKOV3-stable cells expressing Tyr19Phe or WT CDK9. Cells treated with 10 μΜ proteasome inhibitors (MG132 or PS-341) for the indicated time. Data represent two repeats with similar results. **k**,**l**, Expression of ubiquitination and Skp2 examined by WB after IP with FLAG antibody. **k**, SKOV3 cells stably expressing WT or Tyr19Phe CDK9 treated with MG132 (10 μΜ). **l**, SKOV3 cells stably expressing WT CDK9 treated with or without ALK inhibitor (lorlatinib, 0.5 μM) and MG132 (10 μΜ). Data represent two repeats with similar results. Statistical analysis was carried out using the two-tailed, unpaired Student’s *t*-test (**e**) or two-way ANOVA (**f**, **g** and **i**). NS, not significant. ^*^P < 0.05, ^**^P < 0.01, ^***^P < 0.001. Source data

**Extended Data Fig. 7:**
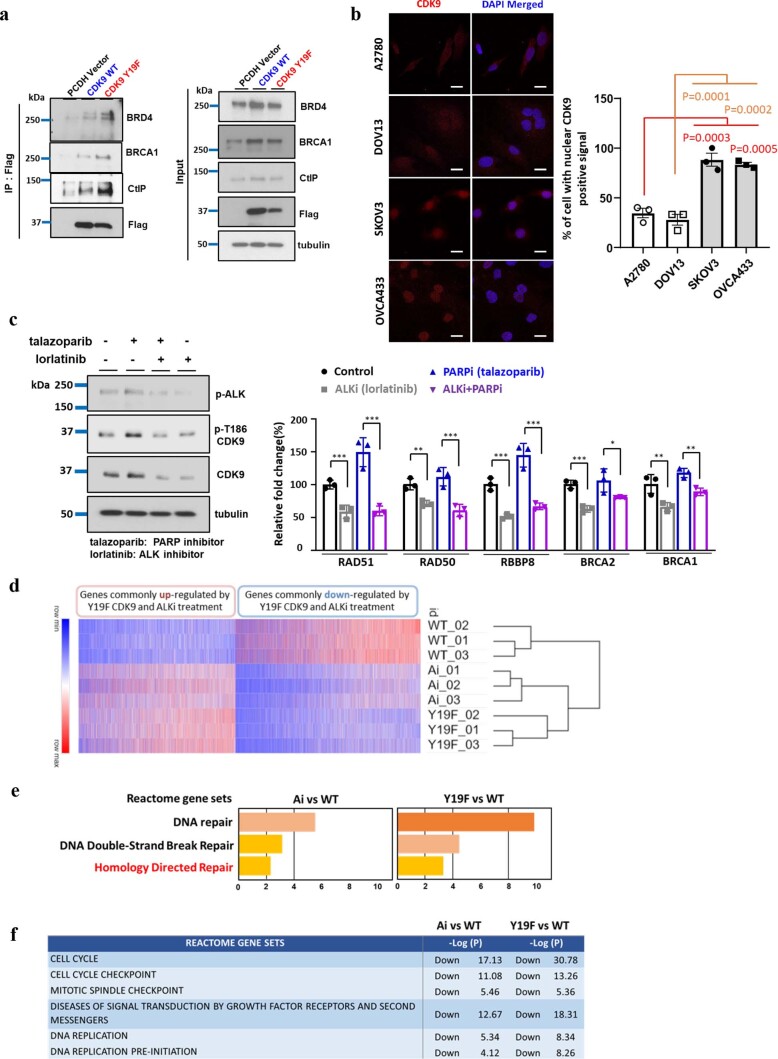
ALK is critical for nuclear localization of CDK9 and transcriptional activates HR repair genes. (**a**) Expression of the indicated proteins in 293 T cells expressing Y19F and WT CDK9 was examined by WB after IP with FLAG antibody. Data are representative of two repeats with similar results. (**b**) Representative images of CDK9 with DAPI staining (left panel) and quantification of cells with nuclear CDK9 positive signal (right panel) in PARPi resistant (OVCA433 and SKOV3) or PARPi sensitive (A2780 and DOV13) ovarian cancer cells. Bar, 20 µm. Data are represented as mean ± SEM of N = 3 independent experiments. One-way ANOVA analysis, A2780 vs SKOV3: ***P = 0.0003, A2780 vs OVCA433: ***P = 0.0005, DOV13 vs SKOV3: ***P = 0.0001, DOV13 vs OVCA433: ***P = 0.0002. (**c**) Western blot analysis of indicated proteins (left) and real-time PCR analysis of indicated genes (right) in PARPi-resistant SKOV3 cells treated with 0.5 μM PARPi (talazoparib) or 0.5 μM ALK inhibitor (lorlatinib), either alone or in combination, for 24 hours. Data are represented as mean ± SD of N = 3 independent experiments. Con vs ALKi: ***P = 0.0001 in RAD51, **P = 0.0079 in RAD50, ***P < 0.0001 in RBBP8, ***P = 0.0005 in BRCA2, **P = 0.0013 in BRCA1; PARPi vs PARPi+ALKi: ***P < 0.0001 in RAD51, ***P < 0.0001 in RAD50, *P = 0.0241in RBBP8, **P = 0.0097in BRCA2, *P = 0.0122 in BRCA1,two-way ANOVA analysis. (**d**) Transcriptome analysis of treatment with ALK inhibitor and Y19F CDK9 mutant. Heatmap of genes that were up- or down-regulated in both WT CDK9-ALKi (ALKi treatment) and Y19F-mutant CDK9 (Y19F) cells relative to WT CDK9 (WT) cells. (**e**) Metascape results showing gene sets related to DNA repair in ALKi treated WT CDK9 cells and Y19F-mutant CDK9 cells relative to WT CDK9 cells. Pre-ranked gene lists for pathway enrichment analysis were generated using the results (P-value) from DEseq2. (**f**) Metascape report of other Reactome gene sets that were ranked before DNA repair related gene sets and enriched in genes down-regulated in both WT CDK9-ALKi and Y19F-mutant CDK9 cells. Metascape utilizes the well-adopted hypergeometric test and Benjamini-Hochberg p-value correction algorithm for statistical analysis. WT, wild-type; Ai, ALK inhibitor treatment; Y19F, Tyr19Phe mutation. Source data

Next, we were interested in determining the functional importance of p-Tyr19-CDK9. Phosphorylation of Thr186 within the T-loop of CDK9 is important for its kinase activity. In addition, previous studies suggest that phosphorylation of CDK9 at Thr186 induces formation of the positive transcription elongation factor b (P-TEFb) with cyclin T and P-TEFb-dependent Pol II Ser2 phosphorylation to activate gene transcription^40–43^.To determine whether p-Tyr19 affects CDK9 kinase activity and transcriptional activity by regulating the formation of P-TEFb complex and phosphorylation of Thr186 CDK9 and Pol II Ser2, we performed IP–WB of lysates from stable cell lines expressing WT or Tyr19Phe CDK9. It is interesting that the p-Thr186 CDK9 was attenuated in Tyr19Phe CDK9-expressing cells compared with WT CDK9 cells, indicating that p-Tyr19 is important for p-Thr186-mediated CDK9 kinase activity (Fig. 5a). We further showed that Tyr19Phe CDK9 mutant blocked the binding of cyclin T and phosphorylation of Pol II Ser2, suggesting that p-Tyr19-CDK9 may also regulate the formation of the P-TEFb complex and transcriptional activity of RNA pol II (Fig. 5a). Consistently, ALK inhibition also blocked the binding of cyclin T and phosphorylation of Thr186 CDK9 and Pol II Ser2 when p-Tyr19-CDK9 was inhibited (Fig. 5b), suggesting that ALK is critical for kinase activity of the CDK9- and P-TEFb-mediated transcriptional activity of RNA Pol II. In addition, CDK9 has been shown to directly interact with BRCA1, CtIP and bromodomain-containing 4 (BRD4) to regulate DNA-damage repair. To test whether inhibition of p-Tyr19-CDK9 reduces the interaction between each of these proteins and CDK9, we performed IP–WB of lysates from cells expressing WT or Tyr19Phe CDK9. The results showed that the interaction between each of these proteins and CDK9 was not diminished by blocking p-Tyr19-CDK9 (Extended Data Fig. 7a). As CDK9 has been detected as being activated in the cytoplasm and delivered to the nucleus for promotion of gene transcription after assembly with cyclin T^44,45^, we then tested whether ALK drives CDK9 Tyr19 phosphorylation to facilitate nuclear translocation of CDK9. It is of interest that the results showed that blocking p-ALK and p-Tyr19-CDK9 reduced the nuclear localization of CDK9 (Fig. 5c–e). In addition, we found that nuclear localization of CDK9 was increased in PARP inhibitor-/platinum-resistant cells compared with sensitive cells (Extended Data Fig. 7b). As P-TEFb-dependent Pol II Ser2 phosphorylation is essential for inducing transcriptional activation of DNA-repair pathways, we hypothesized that Tyr19Phe would alter expression of genes, including those involved in HR-repair pathways. Indeed, depletion of CDK9 resulted in downregulation of HR-repair genes with PARP inhibitor treatment (Fig. 5f), and re-expression of WT but not Tyr19Phe CDK9 in CDK9-delepted cells restored the expression of these genes in the HR-repair pathway (Fig. 5f). In addition, ALK inhibition reduced expression of HR-repair genes in cells expressing WT CDK9 (Fig. 5g and Extended Data Fig. 7c) but not in cells re-expressing Tyr19Phe CDK9 (Fig. 5g). The above findings are further supported by results showing that protein levels of HR factors, but not PARP, were reduced by ALK inhibitor and the Tyr19Phe mutant (Fig. 5h). Together, these results suggested that p-Tyr19-CDK9 mediated by ALK increases kinase activity of CDK9, which is critical for its nuclear localization and formation of P-TEFb to transcriptionally activate HR-repair genes via phosphorylation of RNA Pol II Ser2. To further demonstrate the importance of the HR-repair pathway in the network signaling regulated by the ALK–CDK9-Tyr19 axis, we performed RNA-sequencing (RNA-seq) on three groups: (1) cells expressing WT CDK9, (2) cells expressing WT CDK9 treated with an ALK inhibitor and (3) cells expressing Tyr19Phe mutant CDK9. Based on the results from differential gene expression analysis, we observed that the gene set for genes were up- or downregulated in both ALK inhibitor treated WT-CDK9 and Tyr19Phe-mutant CDK9 cells relative to WT CDK9 cells (Extended Data Fig. 7d). We further performed pathway enrichment analysis of downregulated genes with the Reactome gene sets using Metascape. The analysis revealed that gene sets HOMOLOGY DIRECTED REPAIR was enriched in genes downregulated in ALK inhibitor-treated WT CDK9 cells (Extended Data Fig. 7e; Ai, ALK inhibitor versus WT, wild-type: *P* = 0.0041) and Tyr19Phe-mutant CDK9 cells (Extended Data Fig. 7e; Tyr19Phe versus WT, *P* = 0.0004). Other notable gene sets related to DNA-damage response that were enriched in genes downregulated by ALK inhibitor and Tyr19Phe-mutant CDK9 included CELL CYCLE CHECKPOINT, MITOTIC SPINDLE CHECKPOINT and DNA REPLICATION (Extended Data Fig. 7f). It is interesting that PARP1 and HR-repair factors have been shown to repair the DNA damage caused by defects in these pathways. Taken together, these findings suggest that dual inhibition of PARP activity and ALK–Tyr19-CDK9-mediated HR repair is important for accumulation of DNA damage and consequent synthetic lethality.

**Extended Data Fig. 8:**
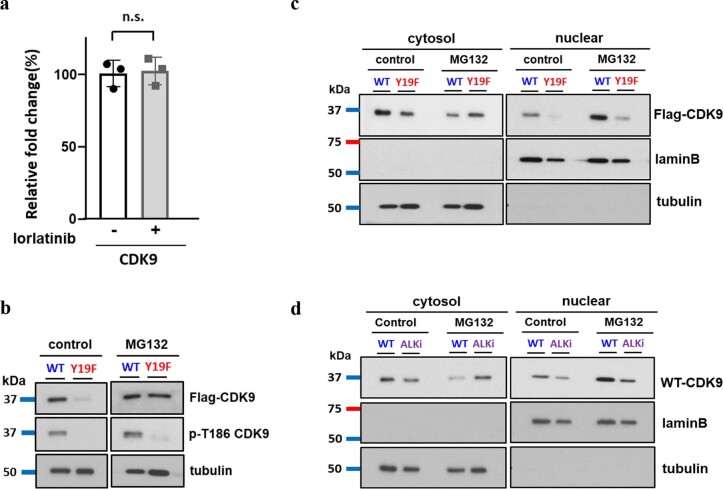
Reduction of CDK9 kinase activity and CDK9 expression within the nucleus is not due to increased degradation. (**a**) Real-time PCR analysis of endogenous CDK9 mRNA expression in SKOV3 cells treated with or without ALK inhibitor. Data are represented as mean ± SD of N = 3 independent experiments. n.s., not significant, two-tailed unpaired t test. (**b, c**) p-T186 expression (**b**) and subcellular localization (**c**) of Flag-tagged CDK9 in cells expressing Y19F and WT CDK9. (**d**) Subcellular localization of CDK9 in cells expressing WT CDK9 treated with or without ALKi. Data are representative of two repeats with similar results. Cells were treated with 10 μΜ proteasome inhibitor (MG132) for 3 h prior to WB analysis (**b–d**). Source data

In parallel, the CDK9 protein level in Tyr19Phe CDK9 mutant cells was lower than that in WT CDK9 cells (Fig. 5a). In addition, although the exogenous CDK9 protein level was decreased by ALK inhibitor (Fig. 5b), the endogenous RNA level of CDK9 was not significantly changed (Extended Data Fig. 8a). To determine whether CDK9 protein stability is affected by p-Tyr19, we treated Tyr19Phe CDK9 and WT CDK9 cells with cycloheximide (CHX). Indeed, CDK9 protein stability was reduced in Tyr19Phe CDK9-expressing cells compared with WT CDK9-expressing cells. Notably, ALK inhibitor treatment also reduced CDK9 protein stability in WT CDK9-expressing cells (Fig. 5i). As Tyr19 is located near the lysine residues, Lys18, Lys21 and Lys24, which are known sites of CDK9 ubiquitination^46,47^, we sought to determine whether phosphorylation of Tyr19 affects the proteasome degradation of CDK9. Indeed, CDK9 protein expression in Tyr19Phe CDK9 cells was restored by treatment with proteasome inhibitors (Fig. 5j). To determine whether the reduction in kinase activity and nuclear localization of Tyr19Phe CDK9 is due to protein degradation, we first assessed the expression of CDK9 and p-Thr186 CDK9 in Tyr19Phe CDK9 and WT CDK9 cells treated with the proteasome inhibitor. Notably, MG132 restored the expression of CDK9, but not p-Thr186 CDK9, in cells expressing Tyr19Phe CDK9, suggesting that the reduction of kinase activity in Tyr19Phe CDK9 cells is not due to increased degradation of CDK9 (Extended Data Fig. 8b). Furthermore, MG132 restored protein levels of Tyr19Phe CDK9 in the cytoplasm but not in the nucleus (Extended Data Fig. 8c). Consistently, MG132 also restored protein levels of CDK9 in the cytoplasm of cells treated with ALK inhibitors, but not in the nucleus (Extended Data Fig. 8d). These results suggest that the blocking of p-ALK and p-Tyr19-CDK9 that reduces the nuclear localization of CDK9 is not due to selective degradation within the nucleus. We further examined the effects of the Tyr19Phe mutation on ubiquitination of CDK9 and demonstrated that ubiquitination was higher in Tyr19Phe CDK9 cells than in WT CDK9 cells (Fig. 5k). In line with the above findings, ALK inhibitor treatment led to an increase in ubiquitination of CDK9 in WT CDK9 cells (Fig. 5l). As Skp2, an E3 ligase, has been reported to promote ubiquitination of CDK9 (ref. ^48^), we sought to determine whether the interaction between Skp2 and CDK9 was affected by ALK-mediated p-Tyr19-CDK9. It is interesting that the Tyr19Phe mutation and ALK inhibition enhanced the interaction between Skp2 and CDK9 (Fig. 5k,l). Together, these data suggest that inhibition of p-ALK and p-Tyr19-CDK9 increases the ubiquitination and degradation of CDK9 by enhancing the binding of Skp2.

### Potential therapeutic strategy targeting PARP1 and ALK

**Fig. 6:**
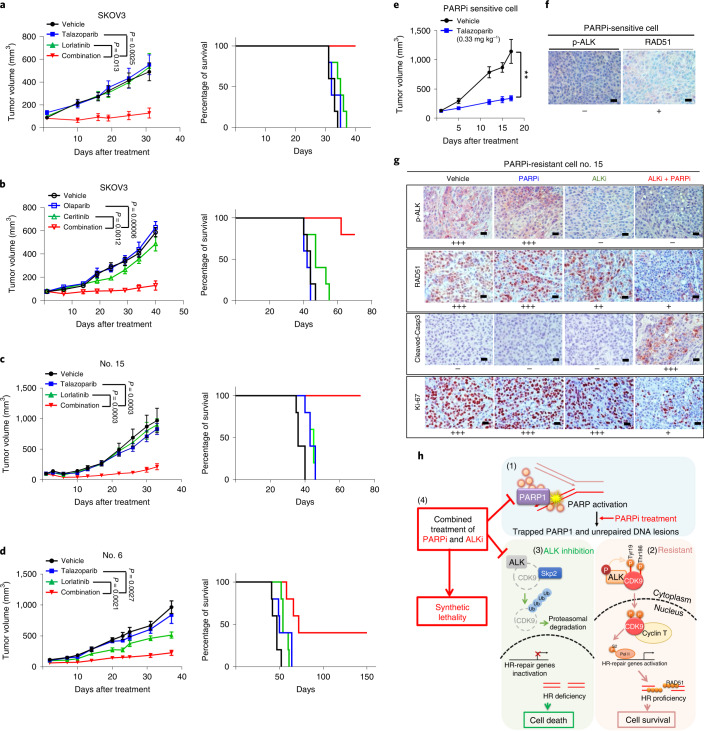
The combination of ALK and PARP inhibitors effectively suppresses tumor growth in vivo. **a**–**d**, Tumor volume and Kaplan–Meier survival curves of mice bearing subcutaneous injected SKOV3 ovarian tumors (**a** and **b**) and orthotopic PARP inhibitor-resistant (acquired resistance) SUM149 tumors (nos. 6 and 15; **c** and **d**). Mice were treated with oral talazoparib (0.33 mg kg^−1^) and lorlatinib (5 mg kg^−1^), either alone or in combination, five times per week (**a**, **c** and **d**), or with oral olaparib (50 mg kg^−1^) and ceritinib (7.5 mg kg^−1^), either alone or in combination, five times per week (**b**). Tumor volume data were reported as mean ± s.e.m. Statistical analysis was carried out using two-tailed, unpaired Student’s *t*-test (*n* = 5 mice in each treatment group). **e**,**f**, Tumor volume curves (**e**) and representative images of IHC staining (**f**) of PARP inhibitor-sensitive SUM149 (parental) tumors from mice with the indicated antibodies. Mice were treated with oral talazoparib (0.33 mg kg^−1^) five times per week. Scale bar, 20 µm. Tumor volume data were reported as mean ± s.e.m. (*n* = 5 mice in each treatment group). Statistical analysis was carried out using the two-tailed, unpaired Student’s *t*-test: ^**^P = 0.0052. **g**, Representative IHC images stained with indicated antibodies in tumor tissues from mice bearing orthotopic PARP inhibitor-resistant (acquired resistance) SUM149 cells. Data represent images of *n* = 3 mice. Scale bar, 20 µm. **h**, Model of PARP inhibitor-resistant mechanism mediated by the ALK–p-Tyr19-CDK9 axis: (1) inactivation of PARP leads to PARP1 trapping on the DNA and increases unrepaired DNA lesions; (2) in PARP inhibitor-resistant cells, p-ALK interacts with and tyrosine phosphorylates CDK9 at Tyr19 to increase the protein stability and kinase activity of CDK9; p-Tyr19-CDK9 regulates formation and nuclear localization of P-TEFb and transcriptionally activates HR-repair genes by phospho-Ser2-RNA Pol II, which in turn contributes to HR proficiency and PARP inhibitor resistance; (3) p-ALK is inhibited by treatment with ALK inhibitors, and the ubiquitination and proteasomal degradation of CDK9 are increased by binding of the E3 ligase Skp2, which in turn blocks the transcription of HR-repair genes and increases PARP inhibitor sensitivity and cell death; and (4) ALK inhibitors induce synthetically lethal PARP inhibitors via induction of HR deficiency. Source data

**Extended Data Fig. 9:**
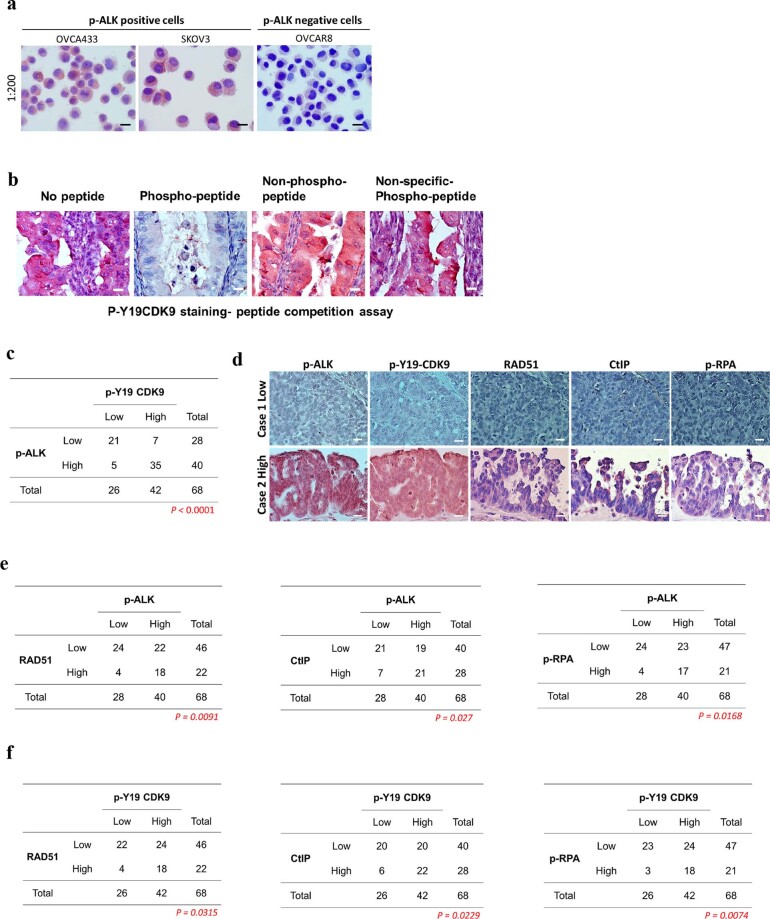
Clinical relevance of ALK activation, p-Y19 CDK9 expression and HR factors in ovarian cancer. (**a**) Characterization of p-ALK antibody in cytospin slides. Data are representative of two repeats with similar results. (**b**) A peptide competition assay was carried out to characterize p-Y19 CDK9 antibody by IHC staining of tumor tissue from ovarian cancer patient. Different peptides as indicated were used to incubate with p-Y19 CDK9 antibody. Data are representative of two repeats with similar results. (**c**) Clinical relevance of p-ALK and p-Y19 CDK9 in human ovarian TMA analysis (Biomax,US, #OV809b). (**d–f**) Representative images (**d**) and quantification of IHC staining for the correlation between HR factors (RAD51, CtIP, or p-RPA) and p-ALK (**e**) or p-Y19 CDK9 (**f**), by human ovarian tissue microarray analysis (Biomax,US, #OV809b). Bar, 20 µm. Statistical analysis was carried out using the two-side Fisher’s exact test. The experiment was performed an additional time with similar results.

**Extended Data Fig. 10:**
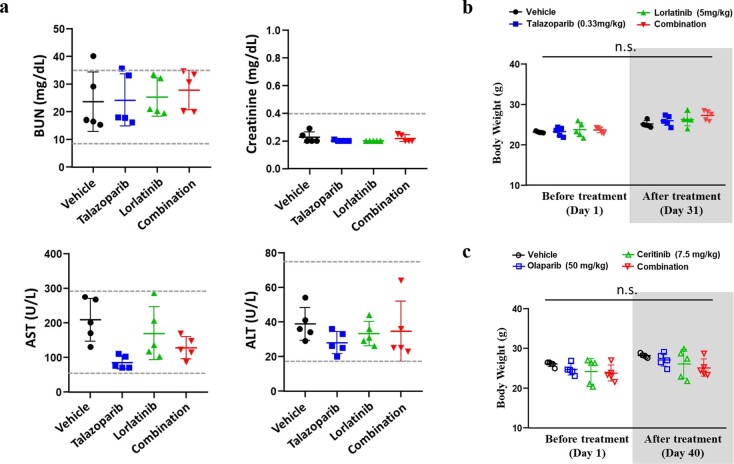
Toxicity assay results and body weight measurements of mice treated with PARP inhibitors (PARPi) and ALK inhibitors. (**a**) Panels of alanine aminotransferase (ALT), aspartate aminotransferase (AST), blood urea nitrogen (BUN) and creatinine in mice bearing PARPi-resistant tumors. Mice were treated with oral talazoparib (0.33 mg/kg) and lorlatinib (5 mg/kg), either alone or in combination, five times per week. (**b, c**) Body weight of mice bearing PARPi-resistant tumors. Mice were treated with oral talazoparib (0.33 mg/kg) and lorlatinib (5 mg/kg), either alone or in combination, five times per week (**b**), or with oral olaparib (50 mg/kg) and ceritinib (7.5 mg/kg), either alone or in combination, five times per week (**c**). N = 5 mice were used in each treatment group. Error bars represent the mean ± SD. Statistical analysis was carried out using the two-way ANOVA analysis with no statistical significance specified as n.s. Source data

Next, we evaluated the clinical relevance of our findings by analysis of p-ALK- and p-Tyr19-CDK9 in tumor tissue from cancer patients (Extended Data Fig. 9a–d). It is of interest that we observed a positive correlation between p-ALK- and p-Tyr19-CDK9 in patients with ovarian cancer (Extended Data Fig. 9c). Furthermore, the HR factors, including RAD51, CtIP and p-RPA, were positively correlated with p-ALK (Extended Data Fig. 9d,e) and p-Tyr19-CDK9 expression (Extended Data Fig. 9d,f). These results suggested that p-Tyr19-CDK9 mediated by ALK is clinically relevant in cancer patients and supports p-ALK–p-Tyr19-CDK9 possibly being potential biomarkers for HR activity and predicting tumor resistance to PARP inhibitors. Next, we evaluated the individual and combined effects of PARP and ALK inhibitors in vivo by utilizing ovarian cancer xenograft models and tumor xenograft models of TNBC with acquired resistance to PARP inhibitors. Mice bearing ovarian cancer xenografts treated with PARP and ALK inhibitors had reduced tumor growth compared with those that received either drug alone (Fig. 6a,b). Similar to our observation in ovarian cancer xenograft models, mice bearing PARP inhibitor-resistant TNBC xenografts (Fig. 6c,d) treated with the combination demonstrated reduced tumor growth compared with those treated with either agent alone. Indeed, PARP inhibitor monotherapy significantly suppressed tumor growth in mice bearing xenografts derived from parental PARP inhibitor-sensitive cells (Fig. 6e), but not cells with acquired resistance to PARP inhibitors (Fig. 6c,d). In both ovarian and breast cancer PARP inhibitor-resistant xenograft models, mice treated with the combination of PARP and ALK inhibitors demonstrated improved survival compared with mice treated with monotherapy (Fig. 6a–d, right panels). Analysis of body weight and biochemical indicators did not suggest increased toxicity in mice treated with the combination of PARP and ALK inhibitors or either drug alone compared with vehicle control (Extended Data Fig. 10). IHC staining of PARP inhibitor-sensitive tumors from mice indicated weak expression of RAD51 with no staining of p-ALK (Fig. 6f), suggesting that HR repair and ALK activity are low in PARP inhibitor-sensitive tumors. In contrast, strong p-ALK and RAD51 expression were detected in PARP inhibitor-resistant tumors from mice treated with vehicle or PARP inhibitor alone (Fig. 6g), suggesting that HR repair and ALK activity are high in PARP inhibitor-resistant tumors. Importantly, the combination of PARP and ALK inhibitors decreased the expression of HR-repair protein, RAD51, and cell proliferation marker, Ki-67, as well as increased expression of the apoptotic marker, cleaved caspase-3 (Fig. 6g). Collectively, these data indicated that ALK is an important therapeutic target for overcoming PARP inhibitor resistance in ovarian cancer and TNBC.

## Discussion

Our present study presents a model (Fig. 6h) showing that ALK contributes to PARP inhibitor resistance by directly phosphorylating CDK9 at Tyr19, thereby increasing the kinase activity and nuclear localization of CDK9 to stabilize the P-TEFb complex, which in turn promotes HR activity through activation of RNA Pol II to turn on HR-repair gene transcription. CDK9 has been shown to have a kinase-dependent role in DNA repair^38^, including HR and replication stress response, which have been implicated as mediators of resistance to cancer therapy such as chemotherapy and PARP inhibitors. In response to replication stress, CDK9 forms a complex with cyclin K and interacts with checkpoint signaling proteins, including the ataxia telangiectasia and Rad3-related protein (ATR), to maintain stability of the replication fork^49^. Notably, CDK9 has been shown to interact and colocalize on DNA-damage sites with BRCA1, a key protein involved in HR^39^. Similar to our observation in cells treated with an ALK inhibitor, CDK9-deficient cells exhibit reduced HR-repair activity and fail to form RAD51 and BRCA1 foci at sites of DNA damage^39^. Of note, CDK9 can also phosphorylate and activate BRD4 (ref. ^50^), a protein that has been recently identified as increasing HR-repair activity by regulating CtIP^51^. In our study, we found that CDK9 transcriptionally activates HR-repair genes in addition to interacting with and regulating key proteins involved in HR. CDK9 is a well-known, transcription-associated CDK which can promote gene transcription by assembling with cyclin T to form the P-TEFb complex and phosphorylating RNA Pol II CTD at Ser2 for active transcription^42^. A recent study demonstrated that inhibition of PARP1-mediated cyclin T PARylation induces the hyperphosphorylation of RNA Pol II CTD by CDK9 (ref. ^52^). It is interesting that our results showed that Tyr19Phe CDK9 failed to phosphorylate RNA Pol II at Ser2, resulting in a HR-deficient phenotype in PARP inhibitor-resistant cells. Our study provided the mechanistic insight to suggest that CDK9 can function as a hub to transduce oncogenic signals from ALK into HR-related transcriptional activity. In so doing, ALK promotes malignant cell survival to protect tumor cells from drug-induced genotoxic stress. Similar to our observation, CDK9 has been shown to interact with a transmembrane receptor, gp130, and transduce interleukin-6 signals into transcription of cellular and viral genes^44^. Our findings also suggest that CDK9-mediated transcriptional activation is a critical downstream signaling axis that enables membrane receptors such as RTK to modulate the DNA-damage response.

PARP inhibitors have limited utility in the treatment of HR-proficient tumors and restoration of HR is a major mechanism of acquired resistance to PARP inhibitors^20^. Thus, there is an urgent need to develop rational combinatorial treatment strategies and identify predictive biomarkers to expand the use of PARP inhibitors to patients with HR-proficient tumors and reverse acquired resistance to PARP inhibitors. In our study, we demonstrated that p-ALK expression is higher in PARP inhibitor-resistant cells compared with PARP inhibitor-sensitive cells. Through functional studies, we further showed that p-ALK regulates HR-repair activity and expression of HR-repair genes, including *BRCA1* and *BRCA2* in PARP inhibitor-resistant cells. Given the key role of the *BRCA1*/*2* complex in RAD51 foci formation on sites of DNA damage to induce HR repair^53^, we measured RAD51 foci formation as a marker of HR activity under several experimental conditions and showed that ALK inhibition reduces RAD51 foci formation in PARP inhibitor-resistant but not PARP inhibitor-sensitive cells. This may be due to the limited p-ALK-dependent transcription of *BRCA1*/*2* and HR activity in PARP inhibitor-sensitive cells, suggesting that the effects of ALK inhibitors on reducing HR activity and HR-repair genes including *BRCA1*/*2* are specific to PARP inhibitor-resistant cells that have high p-ALK expression. Indeed, dual PARP and ALK inhibition led to marked synergistic cell killing effects in vitro and in vivo, providing scientific rationale for inducing a HR-deficient phenotype and consequently (re-)sensitizing PARP inhibitor-resistant tumors to PARP inhibitors via ALK inhibition. Notably, the availability of selective ALK inhibitors currently approved for use in the clinic will facilitate rapid translation of promising combinatorial treatment strategies involving ALK and PARP inhibitors for patients whose tumors have high p-ALK expression, making this a practical approach worthy of further investigation in the clinic. Although there are an increasing number of clinical trials evaluating PARP inhibitors as monotherapy or in combination with other agents in several different cancer types^54^, the lack of predictive biomarkers in this space have substantially limited our ability to maximize the therapeutic window for patients in this setting. Although frequently used as a marker of HR deficiency in the clinic, the presence of germline *BRCA1*/*2* mutations does not consistently predict response to PARP inhibitors^55^. Furthermore, despite the notable effort invested in clinical trials of combinatorial therapeutic strategies involving PARP inhibitors and other agents targeting proteins in DNA-damage response pathways across many cancer types^34^, this approach is frequently limited by overlapping toxicities of the agents used in combination with PARP inhibitors^56^. Thus, more predictive and functional HR biomarkers with higher accuracy are urgently needed to aid patient selection for treatment with PARP inhibitors. Our study showed that ALK and CDK9 kinase pair and their specific phosphorylated counterparts (p-ALK–p-Tyr19-CDK9) can be utilized to further enhance our ability to select patients whose tumors have a high likelihood of responding to combined PARP and ALK inhibition. In summary, our findings identified not only a predictive biomarker of PARP inhibitor resistance but also a drug target, the oncoprotein ALK, which has limited functionality in normal human adult cells. Thus, the rational combination of ALK and PARP inhibitors holds great promise for expanding the utility of PARP inhibitors to many tumor types with HR proficiency.

## Methods

This present study complies with all relevant ethical regulations. Tumor biospecimens utilized in the present study were taken according to guidelines approved by the Institutional Review Board at the University of Texas MD Anderson Cancer Center (protocol no.: LAB02-187_MODCR0020). Experiments with mice were conducted under the approval of the Institutional Animal Care and Use Committee (IACUC) at the University of Texas MD Anderson Cancer Center (protocol no. 00001250-RN01).

### Cell culture

The SKOV3 (catalog no. HTB-77) and OVCAR3 (catalog no. HTB-161) ovarian cancer cell lines were obtained from American Type Culture Collection (ATCC). The OVCA433 and OVCA420 ovarian cancer cell lines were obtained from A.K.S.’s lab (MD Anderson Cancer Center). SKOV3, OVCAR3, OVCA433 and OVCA420 were cultured in Dulbecco’s modified Eagle’s medium/F12 supplemented with 10% fetal bovine serum (FBS) and 1% antibiotic mixture containing 100 units ml^−1^ of penicillin and 100 mg ml^−1^ of streptomycin (P/S). The DOV13 and A2780 ovarian cancer cell lines were obtained from A.K.S.’s lab and maintained in RPMI medium containing 10% FBS and 1% P/S. The SUM149 (catalog no. CS-07) TNBC cell line was obtained from Asterand Biosciences and maintained in F12K medium containing 5% FBS, 10 mM Hepes, 1 mg ml^−1^ of hydrocortisone, 5 µg ml^−1^ of insulin and 1% P/S. All cell lines were validated by short tandem repeat (STR) DNA fingerprinting using the AmpF_STR identifier kit following the manufacturer’s protocol (Applied Biosystems, catalog no. 4322288). The STR profiles were compared with ATCC fingerprints (ATCC.org) and the Cell Line Integrated Molecular Authentication database v.0.1.200808 (http://bioinformatics.istge.it/clima/)^57^. The PARP inhibitor-resistant TNBC cell lines nos. 6 and 15 were obtained by exposing the SUM149 TNBC cell line to increasing concentrations of talazoparib.

### Chemicals and antibodies

Anti-neoplastic agents used in the present study, including talazoparib (PARP inhibitor), lorlatinib (ALK inhibitor), ceritinib (ALK inhibitor), cisplatin and CDK9-IN-2 (CDK9 inhibitor), were purchased from MedChemExpress. The following antibodies were used for WB: anti-p-ALK (1:500), anti-CDK9 (1:2,000), anti-p-Thr186 CDK9, (1:2,000), anti-p-Rpb1 CTD (1:2,000), anti-cyclin T1 (1:1,000), anti-FLAG tag (1:2,000), anti-PARP (1:1,000), anti-BRD4 (1:2,000), anti-RAD50 (1:3,000), anti-Lamin B1 (1:3,000), anti-BRCA1, anti-CtIP (1:500), anti-p-Tyr (1:5,000) and anti-BRCA2 (1:1,000). The following antibodies were used for IF: anti-RAD51 (1:250) and anti-phospho-histone H2A.X (1:200). The following antibodies were used for IHC: anti-p-ALK (1:200), anti-RAD51 (1:1,000), anti-CtIP (1:10), anti-p-RPA32 (1:100) and anti-p-Tyr19-CDK9 (1:10). Mouse monoclonal antibody against the phosphorylation site of CDK9 at Tyr19 was produced by our lab with a synthetic phosphopeptide: DEVSKP-pY-EKLAKIGQTFGE.

### Receptor tyrosine kinase antibody array

Whole-cell lysates were obtained from PARP inhibitor-/platinum-resistant and PARP inhibitor-/platinum-sensitive ovarian cancer cell lines after a 24-h incubation and applied to a Human Phospho-RTK Array Kit (R&D Systems, catalog no. ARY001B) following the manufacturer’s instructions. In brief, 300 μg of protein from each sample was applied to the nitrocellulose array membranes with capture and control antibodies spotted in duplicate. A pan-p-Tyr antibody conjugated to horseradish peroxidase was then used to detect phosphorylated tyrosines by chemiluminescence. Signal intensities on the membranes were quantified using the image analysis software AlphaEaseFC (Alpha Innotech).

### Western blotting

Cells were washed with cold phosphate-buffered saline (PBS) containing protease inhibitors (bimake.com) and phosphatase inhibitors (biotool.com) before harvesting. Harvested cells were lysed in radioimmunoprecipitation (RIPA) buffer (20 mM Tris-HCl, pH 7.5, 150 mM NaCl, 1 mM Na_2_EDTA, 1 mM (ethylenebis(oxonitrilo))tetra-acetate (EGTA), 1% NP-40, 1% sodium deoxycholate, 2.5 mM sodium pyrophosphate, 1 mM β-glycerophosphate, 1 mM Na_3_VO_4_ and 1 µg ml^−1^ of leupeptin) with protease and phosphatase inhibitors. The total protein concentration in whole-cell lysates was determined using the Pierce BCA protein assay kit (Fisher, catalog no. PI-23227) according to the manufacturer’s protocol. Sample buffer was then added to the whole-cell lysates. Proteins from each sample (10–40 µg) were separated in an 8% or 10% bis–tris sodium dodecylsulfate (SDS)–poly(acrylamide) gel electrophoresis gel and transferred to a poly(vinylidene difluoride) (PVDF) membrane (Millipore). After blocking with 5% fat-free dry milk or bovine serum albumin (BSA), primary antibodies were incubated with the PVDF membranes in 5% BSA overnight at 4 °C. Membranes were washed in TBS-T (50 mM Tris-Cl, pH 7.5, 150 mM NaCl and 0.05% Tween-20) and hybridized with appropriate secondary antibodies in 5% fat-free dry milk for 45–60 min at room temperature and imaged using ECL reagents (BioRad Laboratories). Image acquisition and band intensity quantification were performed using an Odyssey infrared imaging system.

### Immunoprecipitation

Whole-cell lysates were prepared in an IP buffer (25 mM Tris, pH 7.4, 150 mM NaCl, 0.1% NP-40, 1 mM EDTA and 10% glycerol) with protease and phosphatase inhibitors. After overnight incubation at 4 °C with 1 µg of primary antibody or immunoglobulin G control, protein G or protein A–agarose beads were added and the sample was incubated at 4 °C for 3–4 h before washing with an IP buffer. Protein expression was then detected by WB as described in the previous section.

### Cell viability assay

Cells were seeded in a 96-well plate and incubated overnight before treatment with the respective inhibitors for 4–6 d. Cell viability was assessed using the 3-(4,5-dimethylthiazol-2-yl)-2,5-diphenyltetrazolium bromide (MTT) assay. After incubation with 10% MTT (5 mg ml^−1^ in PBS) for 1.5–3 h, dimethylsulfoxide was added to dissolve the water-insoluble purple precipitate. Absorbance of the resulting solution in individual wells was measured at 595 nm, with a reference wavelength of 650 nm, using a BioTek Synergy Neo multi-mode reader. The Chou–Talalay method^58^ was used to calculate the CI using Compusyn software (http://www.combosyn.com).

### Colony formation assay

Cells were plated on 24-well plates. After overnight incubation, cells were treated with various inhibitors for 15 d (inhibitor-containing medium was replaced every 3 d). At the end of 15 d, colonies were fixed and stained with 0.5% Crystal Violet, washed, dried and imaged. Crystal Violet was removed from colonies using 33% acetic acid and absorbance was measured at 540 nm. The Chou–Talalay method^58^ was used to calculate the CI using Compusyn software (http://www.combosyn.com).

### DR–GFP reporter assay

To assess HR activity, U2OS direct repeat–green fluorescent protein (DR–GFP) cells were transfected with I-SceI expression plasmid and treated with talazoparib (50 nM) and lorlatinib (0.5 μM), either alone or in combination, for 48 h. After treatment, cells were trypsinized, washed using PBS with 2% FBS, stained with Ghost Dye Violet 510 (Tonbo Bioscience, catalog no. 130870; 1:200), and suspended in FACS buffer (2 mM EDTA and 2% FBS in PBS). Cell suspensions were then analyzed by BD FACSCanto II cytometer and data were acquired using the BD FACSDiva v.8.0.2 software and processed using FlowJo v.10.7.1 (BD Biosciences). Cell population data were collected on a debris exclusion gate. Live cells were gated by excluding Ghost Dye (Violet 510)-positive cells and further quantified by FITC to identify GFP-positive cell populations. Gating strategy used for flow cytometric analysis is provided in Extended Data Fig. 3f,g.

### IF staining

Cells were treated with talazoparib (50 nM) and lorlatinib (0.5 μM), either alone or in combination, for 48 h. After incubation with 0.01% MMS for 1.5 h, cells were placed in fresh medium for 3 h. To label S-phase cells, the Click-iT EdU Alexa Fluor-488 imaging Kit (Invitrogen, catalog no. C10337) was used according to the manufacturer’s recommendations for EdU staining. Briefly, the cells were incubated with 10 µM EdU for 30 min and then fixed with paraformaldehyde, permeabilized in 0.5% Triton X-100/PBS and washed with PBS. After incubation with freshly prepared Click-iT reaction cocktail for 30 min, cells were then washed with PBS + 3% BSA 3× and incubated in a blocking buffer (5% BSA in PBS) at room temperature for 1 h. After blocking, cells were incubated overnight with primary antibodies against RAD51 (GeneTex, catalog no. GTX100469; 1:250), BRCA1 (Cell Signaling, catalog no. 9010; 1:100), CtIP (Santa Cruz Biotechnology, catalog no. sc-271339; 1:100) or γH2AX (Millipore Sigma, catalog no. 05-636; 1:250) with 5% BSA in PBS at 4 °C. Secondary antibodies conjugated to Alexa Fluor-594 and -488 (Life Technologies, catalog no. A-21203 or A-21208; 1:500) were used to visualize the primary antibody. Cells were counterstained with mounting medium containing DAPI (Life Technologies, catalog no. P36941). Fluorescent images of the cells were acquired using an LSM 710 confocal microscope (Carl Zeiss). Based on the number of foci identified in the nucleus, RAD51-, γH2AX- or BRCA1-positive cells were defined as cells with >10 foci per cell. CtIP- or 53BP1-positive cells were defined as cells with >3 foci per cell (Extended Data Fig. 4e).

### In vitro kinase assay

The expression of the recombinant GST–CDK9 WT and GST–CDK9 mutant (Tyr19Phe/Tyr92Phe/Tyr185Phe/Tyr282Phe) was induced in *Escherichia coli* (BL21) using isopropyl β-d-1-thiogalactopyranoside and purified with glutathione agarose beads. The purified recombinant proteins were then incubated with kinase-active ALK recombinant protein (Abcam, catalog no. ab187246) and 0.2 mM ATP in a kinase buffer (5 mM MgCl_2_, 5 mM MnCl_2_, 50 μM Na_3_VO_4_, 50 mM Hepes, pH 7.4 and 5 mM dithiothreitol) at 30 °C for 30 min. Termination of the kinase reaction was achieved by the addition of SDS sample buffer and heating at 100 °C for 10 min. WB with a p-Tyr antibody (no. 05-321, 4G10) was then performed to determine the extent of tyrosine phosphorylation.

### RT–qPCR

Total RNA was extracted from SKOV3 cells using the RNeasy Mini kit (QIAGEN). Complementary DNA was synthesized using the Applied Biosystems High-Capacity cDNA Reverse Transcription Kit (Thermo Fisher Scientific). All PCR reactions were performed using the following primers (5′ to 3′):

RAD51-F: CGACTCTCCCTGTCTTCCTG; RAD51-R: TTTCCCGGAAGCTTTATCCT

RAD50-F: CTTGGATATGCGAGGACGAT; RAD50-R: CCAGAAGCTGGAAGTTACGC

RBBP8-F: GCAGACAGTTTCTCCCAAGC; RBBP8-R: TGCCCAAGCAGTTTTCTTCT

BRCA1-F: GGTGGTACATGCACAGTTGC; BRCA1-R: TGACTCTGGGGCTCTGTCTT

BRCA2-F: AGCTCTTCACCCTGCAAAAA; BRCA2-R: CCAATGCCTCGTAACAACCT

GAPDH-F: CGACCACTTTGTCAAGCTCA; GAPDH-R: AGGGGTCTACATGGCAACTG. Quantitative PCR with reverse transcription (RT–qPCR) acquisition was captured using BioRad CFX96.

### Transcriptome analysis by RNA-seq

Total RNA was extracted from SKOV3 cells using the RNeasy Mini kit (QIAGEN). RNA-seq was performed by NOVOGENE (University of California, Davis) and mapped to the human reference genome (hg38). All RNA-seq data were deposited in the Gene Expression Omnibus (GEO) data repository with accession no. GSE189695. The heatmap was generated using Morpheus (https://software.broadinstitute.org/morpheus). Pathway enrichment analysis with the Reactome gene sets was performed by Metascape^59^. Preranked gene lists for pathway enrichment analysis were generated using the results (*P* value) from DEseq2.

### Animal studies

All animal studies were performed in accordance with guidelines approved by the MD Anderson IACUC (protocol no. 00001250-RN01). Mice were maintained at an ambient temperature of 21.1 ± 1 °C and relative humidity 30–70% under a 12-h:12-h light:dark cycle. All mice were scheduled for euthanasia once the tumor volume had reached 1,500 m^3^, as indicated in the IACUC protocols. The maximal tumor size of all mice used in the present study did not exceed 1,500 mm^3^_._ For ovarian tumor xenografts, 1.5 × 10^6^ ovarian cancer cells were suspended in a 1:1 mixture of PBS and Matrigel before implantation by direct subcutaneous injection into the flanks of 6- to 8-week-old female nude mice. For TNBC tumor xenografts, 2 × 10^6^ PARP inhibitor-resistant SUM149 TNBC (nos. 6 and 15) or PARP inhibitor-sensitive SUM149 TNBC cells were suspended in a 1:1 mixture of PBS and Matrigel and injected into the mammary fat pad of 6- to 8-week-old female nude mice. Once the tumor volume had reached between 75 and 150 mm^3^, tumor-bearing mice were treated daily with vehicle control (0.5% hydroxypropylmethylcellulose and 0.2% Tween-80, oral gavage), talazoparib (0.333 mg kg^−1^, oral gavage), lorlatinib (5 mg kg^−1^, oral gavage) or the combination of talazoparib and lorlatinib. Five mice were used in each treatment group. The tumor volume was measured every 3–5 d using the following formula: *v* = (*l* × *w*^2^)/2, where *v* is volume, *l* length and *w* weight.

### Patient tissue sample and IHC staining

Use of human tumor tissue specimens followed the guidelines approved by the Institutional Review Board at MD Anderson. Written informed consent was obtained from all patients. By utilizing all available tumor biospecimens, self-selection bias was minimized in the present study. Age and gender were not treated as covariates in the present study. For IHC staining, the tissue specimens were incubated with primary antibodies against p-ALK, p-Tyr19-CDK9, Ki-67, c-caspase-3, RAD51, CtIP or p-RPA, followed by detection with biotin-conjugated secondary antibody and avidin peroxidase, and then visualized using aminoethyl carbazole chromogen. Based on the intensity of staining in sections, the staining intensity was classified into four groups: strong (score ≥ 2), moderate (1 ≤ score < 2), weak (0.5 ≤ score < 1) and negative (0). Cases with weak or negative staining intensity were categorized as having low expression of the indicated protein and those with moderate or strong staining intensity were categorized as having high expression of the indicated protein.

### Statistics and reproducibility

No statistical method was used to predetermine sample size, but our sample sizes are similar to those reported in previous publications^22–24^. No data points and no animals were excluded from the analyses. Data collection and analysis were not performed blinded to the conditions of all experiments. The IHC experiment was performed by the pathologists without any information about patient tissue. Blinding was not used for animal works because the investigators needed to know the treatment groups to perform inhibitor treatment. Blinding was not applicable to the rest of the other in vitro experiments because the same investigator was doing group allocation during data collection and/or analysis. For in vitro experiments, cells were randomly allocated into control and experimental groups. For in vivo experiments, age- and sex-matched mice were randomized into control and experimental groups before tumor size measurement and inhibitor treatment. Data distribution was assumed to be normal but this was not formally tested. The following representative experiments were repeated the indicated the number of times with similar results: the experiments in Figs. 3a,e–j and 5a,b–d,h and Extended Data Figs. 1a–c, 4a, 5b,g,i and 7a,c were repeated twice with similar results. Figure 6f,g shows representative images of *n* = 3 mice. Data are reported as mean ± s.d. or s.e.m. as stated. Statistical analyses were performed with GraphPad Prism 8.0. The difference between groups was compared using the two-tailed, unpaired Student’s *t*-test or analysis of variance (ANOVA) analysis. Two-sided Fisher’s exact analysis was performed to analyze IHC data. A *P* < 0.05 was considered to be statistically significant. ^*^P < 0.05, ^**^P < 0.01, ^***^P < 0.001.

### Reporting summary

Further information on research design is available in the Nature Research Reporting Summary linked to this article.

## Supplementary information

Reporting Summary

## Data Availability

RNA-seq data that support the findings of the present study have been deposited in the GEO under accession no. GSE189695. All data supporting the present study are available within the article and supplementary information files. Source data are provided with this paper.

## Source data

Source Data Fig. 1 Statistical source data.
Source Data Fig. 2 Statistical source data.
Source Data Fig. 3 Statistical source data.
Source Data Fig. 3 Unprocessed western blots.
Source Data Fig. 4 Statistical source data.
Source Data Fig. 5 Statistical source data.
Source Data Fig. 5 Unprocessed western blots.
Source Data Fig. 6 Statistical source data.
Source Data Extended Data Fig. 1 Statistical source data.
Source Data Extended Data Fig. 1 Unprocessed western blots.
Source Data Extended Data Fig. 3 Statistical source data.
Source Data Extended Data Fig. 4 Statistical source data.
Source Data Extended Data Fig. 4 Unprocessed western blots.
Source Data Extended Data Fig. 5 Statistical source data.
Source Data Extended Data Fig. 5 Unprocessed western blots.
Source Data Extended Data Fig. 7 Statistical source data.
Source Data Extended Data Fig. 7 Unprocessed western blots.
Source Data Extended Data Fig. 8 Statistical source data.
Source Data Extended Data Fig. 8 Unprocessed western blots.
Source Data Extended Data Fig. 10 Statistical source data.

## Code availability

Compusyn (http://www.combosyn.com) open-source code was used for analyses performed in this research. The commercial code GraphPad Prism 8 (https://www.graphpad.com/scientific-software/prism) was used for data analysis. Zeiss Zen software (https://www.zeiss.com/microscopy/int/products/microscope-software/zen.html) was used with a Zeiss LSM 710 confocal microscope for IF staining imaging.
