# Supplementary material for: Targeting the ALK–CDK9-Tyr19 kinase cascade sensitizes ovarian and breast tumors to PARP inhibition via destabilization of the P-TEFb complex
Source: Nat Cancer. 2022 Oct 17;3(10):1211–27. doi: 10.1038/s43018-022-00438-2 (PMC9586872; doi:10.1038/s43018-022-00438-2)
Supplement: Supplementary file 1 — Reporting Summary [file 43018_2022_438_MOESM1_ESM.pdf]

nature portfolio

Corresponding author(s): Dr. Mien-Chie Hung
Dr. Dihua Yu

Last updated by author(s): Aug 19, 2022

Reporting Summary

Nature Portfolio wishes to improve the reproducibility of the work that we publish. This form provides structure for consistency and transparency in reporting. For further information on Nature Portfolio policies, see our Editorial Policies and the Editorial Policy Checklist.

Statistics

For all statistical analyses, confirm that the following items are present in the figure legend, table legend, main text, or Methods section.

n/a	Confirmed	
☐	☒	The exact sample size (n) for each experimental group/condition, given as a discrete number and unit of measurement
☐	☒	A statement on whether measurements were taken from distinct samples or whether the same sample was measured repeatedly
☐	☒	The statistical test(s) used AND whether they are one- or two-sided<br>*Only common tests should be described solely by name; describe more complex techniques in the Methods section.*
☒	☐	A description of all covariates tested
☒	☐	A description of any assumptions or corrections, such as tests of normality and adjustment for multiple comparisons
☐	☒	A full description of the statistical parameters including central tendency (e.g. means) or other basic estimates (e.g. regression coefficient) AND variation (e.g. standard deviation) or associated estimates of uncertainty (e.g. confidence intervals)
☐	☒	For null hypothesis testing, the test statistic (e.g. F, t, r) with confidence intervals, effect sizes, degrees of freedom and P value noted<br>*Give P values as exact values whenever suitable.*
☒	☐	For Bayesian analysis, information on the choice of priors and Markov chain Monte Carlo settings
☒	☐	For hierarchical and complex designs, identification of the appropriate level for tests and full reporting of outcomes
☒	☐	Estimates of effect sizes (e.g. Cohen's d, Pearson's r), indicating how they were calculated

Our web collection on statistics for biologists contains articles on many of the points above.

Software and code

Policy information about availability of computer code

Data collection	GraphPad Prism 9, Compusyn (2005 PC windows) , BD FACSDiva v8.0.2
Data analysis	GraphPad Prism 9, Compusyn (2005 PC windows) , BD FACSDiva v8.0.2, FlowJo v10.7.1 software (BD Biosciences), DEseq2, Metascape, Morpheus

For manuscripts utilizing custom algorithms or software that are central to the research but not yet described in published literature, software must be made available to editors and reviewers. We strongly encourage code deposition in a community repository (e.g. GitHub). See the Nature Portfolio guidelines for submitting code & software for further information.

Data

Policy information about availability of data

All manuscripts must include a data availability statement. This statement should provide the following information, where applicable:

- Accession codes, unique identifiers, or web links for publicly available datasets
- A description of any restrictions on data availability
- For clinical datasets or third party data, please ensure that the statement adheres to our policy

RNA–seq data that support the findings of this study have been deposited in the Gene Expression Omnibus (GEO) under accession code GSE189695. Source data files for all figures are provided with this paper. All data supporting this study are available within the article and supplementary information files.

Human research participants

Policy information about studies involving human research participants and Sex and Gender in Research.

Reporting on sex and gender	Sex and gender-based analyses were not performed because this is a study focused on breast and ovarian cancer which overwhelmingly involves female subjects.
Population characteristics	Tumor biospecimens utilized in this study were obtained from patients with ovarian cancer treated with PARP inhibitor-based therapy on an IRB-approved clinical trial at The University of Texas MD Anderson Cancer Center.
Recruitment	All available tumor biospecimens from patients with ovarian cancer enrolled on this clinical trial were utilized in this study, according to guidelines approved by the Institutional Review Board at The University of Texas MD Anderson Cancer Center. Written informed consent was obtained from all patients. By utilizing all available tumor biospecimens, self-selection bias was minimized in this study.
Ethics oversight	Tumor biospecimens utilized in this study was performed according to guidelines approved by the Institutional Review Board at The University of Texas MD Anderson Cancer Center (protocol number: LAB02-187_MODCR0020).

Note that full information on the approval of the study protocol must also be provided in the manuscript.

Field-specific reporting

Please select the one below that is the best fit for your research. If you are not sure, read the appropriate sections before making your selection.

☒ Life sciences ☐ Behavioural & social sciences ☐ Ecological, evolutionary & environmental sciences

For a reference copy of the document with all sections, see nature.com/documents/nr-reporting-summary-flat.pdf

Life sciences study design

All studies must disclose on these points even when the disclosure is negative.

Sample size	No statistical methods were used to predetermine sample sizes, but our sample sizes are similar to those reported in previous studies (ref 22-24 in the manuscript).
Data exclusions	No data points and no animals were excluded from the analyses.
Replication	Reproducibility of results were confirmed by repeating the experiments independently. The precise number of repeats is provided in the Methods section or in the Figure legends.
Randomization	For in vitro experiments, cells were randomly allocated into control and experimental groups. For in vivo experiments, age and sex-matched mice were randomized into control and experimental groups prior to tumor size measurement and inhibitor treatment.
Blinding	IHC experiment was performed by the pathologists without any information about patient tissue. Blinding was not used for animal works because the investigators needed to know the treatment groups in order to perform inhibitor treatment. Blinding was not applicable to the rest of other in vitro experiments (e.g. Western blotting and flow cytometric analysis) because the same investigator was doing group allocation during data collection and/or analysis.

Reporting for specific materials, systems and methods

We require information from authors about some types of materials, experimental systems and methods used in many studies. Here, indicate whether each material, system or method listed is relevant to your study. If you are not sure if a list item applies to your research, read the appropriate section before selecting a response.

Materials & experimental systems

n/a	Involved in the study
☐	☒ Antibodies
☐	☒ Eukaryotic cell lines
☒	☐ Palaeontology and archaeology
☐	☒ Animals and other organisms
☒	☐ Clinical data
☒	☐ Dual use research of concern

Methods

n/a	Involved in the study
☒	☐ ChIP-seq
☐	☒ Flow cytometry
☒	☐ MRI-based neuroimaging

Antibodies

Antibodies used

The following antibodies were used for Western blotting:
Anti-phospho-ALK (Cell Signaling Technology, catalog no. 3341, lot no. 7; 1:500)
Anti-CDK9 (C12F7, Cell Signaling Technology, catalog no. 2316, lot no. 7; 1:2000)
Anti-Phospho-T186-CDK9, (Cell Signaling Technology, catalog no. 2549, lot no. 2; 1:2000)
Anti-Phospho-Rpb1 CTD (E1Z3G, Cell Signaling Technology, catalog no. 13499, lot no. 1; 1;2000)
Anti-Cyclin T1 (D1B6G, Cell Signaling Technology, catalog no. 81464, lot no. 1; 1:1000)
Anti-Flag tag (Cell Signaling Technology, catalog no. 2368, lot no. 12; 1:2000)
Anti-PARP (46D11, Cell Signaling Technology, catalog no. 9532, lot no. 9; 1:1000)
Anti-BRD4 (E2A7X, Cell Signaling Technology, catalog no. 13440, lot no. 9; 1:2000)
Anti-RAD50 (Cell Signaling Technology, catalog no. 3427, lot no. 2; 1:3000)
Anti-Lamin B1 (C-5, Santa Cruz Biotechnology, catalog no. sc-365962, lot no. 00092016; 1: 3000)
Anti-BRCA1 (D-9, Santa Cruz Biotechnology, catalog no. sc-6954, lot no. G2821; 1: 500)
Anti-CtIP (D-4, Santa Cruz Biotechnology, catalog no. sc-271339, lot no. C0320; 1:500)
Anti-Phosphotyrosine (4G10, Millipore-Sigma, catalog no. 05-321, lot no. 3507846; 1:5000)
Anti-BRCA2 (Bethyl Laboratories, catalog no. A303-434A, lot no. 3; 1:1000).

The following antibodies were used for Immunofluorescence:
Anti-RAD51 (N1C2, GeneTex, catalog no. GTX100469, lot no. 42711; 1:250)
Anti-phospho-Histone H2A.X (JBW301, Millipore-Sigma, catalog no. 05-636, lot no. 3108494; 1:200)
Anti-BRCA1 (D-9, Santa Cruz Biotechnology, catalog no. sc-6954, lot no. G2821; 1: 100)
Anti-CtIP (D-4, Santa Cruz Biotechnology, catalog no. sc-271339, lot no. C0320; 1:10)

The following antibodies were used for IHC:
Anti-Phospho-ALK (Invitrogen, catalog no. PA5-40168, lot no. WF3298953A; 1:200)
Anti-RAD51 (N1C2, GeneTex, catalog no. GTX100469, lot no. 42711; 1:1000)
Anti-CtIP (D-4, Santa Cruz Biotechnology, catalog no. sc-271339, lot no. C0320; 1:10)
Anti-Phospho-RPA32 (Bethyl Laboratories, catalog no. A300-245A, lot no. 8; 1:100)
Anti-phospho-Y19-CDK9 (lot no. MCH-01, 1:10); Mouse monoclonal antibody against the phosphorylation site of CDK9 at Tyr 19 was produced with a synthetic phosphopeptide: DEVSKP-pY-EKLAKIGQTFGE.

Validation

All of the antibodies are validated by manufacturer or in the manuscript.

Anti- Phospho-ALK (Tyr1604), Cell Signaling Technology, catalog no. 3341, lot no. 7, Western blotting, https://www.cellsignal.com/products/primaryantibodies/phospho-alk-tyr1604-antibody/3341?site-search-type=Products&N=4294956287&Ntt=p-alk+tyr+1604&fromPage=plp

Anti-CDK9 (C12F7), Cell Signaling Technology, catalog no. 2316, lot no. 7, Western blotting, https://www.cellsignal.com/products/primary-antibodies/cdk9-c12f7-rabbit-mab/2316?site-search-type=Products&N=4294956287&Ntt=cdk9&fromPage=plp

Anti-Phospho-CDK9 (Thr186), Cell Signaling Technology, catalog no. 2549, lot no. 2, Western blotting, https://www.cellsignal.com/products/primaryantibodies/phospho-cdk9-thr186-antibody/2549?site-search-type=Products&N=4294956287&Ntt=cdk9&fromPage=plp

Anti-Phospho-Rpb1 CTD (Ser2) (E1Z3G), Cell Signaling Technology, catalog no. 13499, lot no. 1, Western blotting, https://www.cellsignal.com/products/primary-antibodies/phospho-rpb1-ctd-ser2-e1z3g-rabbit-mab/13499

Anti-Cyclin T1 (D1B6G), Cell Signaling Technology, catalog no. 81464, Western blotting, lot no. 1, https://www.cellsignal.com/products/primaryantibodies/cyclin-t1-d1b6g-rabbit-mab/81464?site-search type=Products&N=4294956287&Ntt=cdk9&fromPage=plp

Anti-Flag Tag, Cell Signaling Technology, catalog no. 2368, lot no. 12, Western blotting, https://www.cellsignal.com/products/primary-antibodies/dykddddk-tag-antibody-binds-to-same-epitope-as-sigma-s-anti-flag-m2-antibody/2368?site-searchtype=Products&N=4294956287&Ntt=flag&fromPage=plp

Anti-PARP (46D11), Cell Signaling Technology, catalog no. 9532, lot no. 9, Western blotting, https://www.cellsignal.com/products/primary-antibodies/parp-46d11-rabbit-mab/9532

Anti-BRD4 (E2A7X), Cell Signaling Technology, catalog no. 13440, lot no. 9, Western blotting, https://www.cellsignal.com/products/primary-antibodies/brd4-e2a7x-rabbit-mab/13440

Anti-RAD50, Cell Signaling Technology, catalog no. 3427, lot no. 2, Western blotting, https://www.cellsignal.com/products/primary-antibodies/rad50-antibody/3427?_=1656347242181&Ntt=3427&tahead=true

Anti-Lamin B1 (C-5), Santa Cruz Biotechnology, catalog no. sc-365962, lot no. 00092016, Western blotting, https://www.scbt.com/p/lamin-b1-antibody-c-5

Anti-BRCA1 (D-9), Santa Cruz Biotechnology, catalog no. sc-6954, lot no. G2821, Western blotting/ Immunofluorescence, https://www.scbt.com/p/brca1-antibody-d-9?requestFrom=search

Anti-CtIP (D-4), Santa Cruz Biotechnology, catalog no. sc-271339, lot no. C0320, Western blotting/ Immunofluorescence, https://www.scbt.com/p/ctipantibody-d-4?requestFrom=search

Anti-phospho-Histone H2A.X (Ser139) (JBW301), Millipore-Sigma, catalog no. 05-636, lot no. 3108494, Immunofluorescence, https://www.emdmillipore.com/US/en/product/Anti-phospho-Histone-H2A.X-Ser139-Antibody-clone-JBW301,MM_NF-05-636

Anti-Phosphotyrosine (4G10), Millipore-Sigma, catalog no. 05-321, lot no. 3507846, Western blotting, https://www.emdmillipore.com/US/en/product/Anti-Phosphotyrosine-Antibody-clone-4G10,MM_NF-05-321

Anti-BRCA2, Bethyl Laboratories, catalog no. A303-434A, lot no. 3, Western blotting, https://www.thermofisher.com/antibody/product/BRCA2-Antibody-Polyclonal/A303-434A

Anti-Phospho-RPA32 (Ser4, Ser8), Bethyl Laboratories, catalog no. A300-245A, lot no. 8, Immunohistochemistry, https://www.thermofisher.com/antibody/product/Phospho-RPA32-Ser4-Ser8-Antibody-Polyclonal/A300-245A

Anti-RAD51 (N1C2), GeneTex, catalog no. GTX100469, lot no. 42711, Immunofluorescence/ Immunohistochemistry, https://www.genetex.com/Product/Detail/Rad51-antibody-N1C2/GTX100469

Anti-pY19-CDK9 mouse monoclonal antibody (lot no. MCH-01) was validated in the Extended Data Figure 9b.

Eukaryotic cell lines

Policy information about cell lines and Sex and Gender in Research

Cell line source(s)	The SKOV3 (catalog no. HTB-77) and OVCAR3 (catalog no. HTB-161) ovarian cancer cell lines were obtained from American Type Culture Collection (ATCC). The SUM149 (catalog no. CS-07) TNBC cell line was obtained from Asterand Biosciences (Detroit, MI). The PARPi-resistant TNBC cell lines #6 and #15 were obtained by exposing the SUM149 TNBC cell line to increasing concentrations of talazoparib. The OVCA433, DOV13, A2780 and OVCA420 ovarian cancer cell lines were obtained from Dr. Anil K. Sood lab (MD Anderson Cancer Center).
Authentication	Cells were authenticated by short tandem repeat DNA finger printing.
Mycoplasma contamination	They were negative for mycroplasma.
Commonly misidentified lines (See ICLAC register)	No commonly misidentified cell lines were used in this study.

Animals and other research organisms

Policy information about studies involving animals; ARRIVE guidelines recommended for reporting animal research, and Sex and Gender in Research

Laboratory animals	The 6- to 8-week-old, female nude mice were used in this study. Mice were maintained at an ambient temperature of 70 ± 2°F and relative humidity of 30–70% under a 12-h light/12-h dark cycle. All mice were scheduled for euthanasia once tumor volume had reached 1,500m3, as indicated in the IACUC protocols. The maximal tumor size of all mice used in this study was not exceeded 1,500 mm3.
Wild animals	No wild animals were used in the study.
Reporting on sex	Only female mice were used in this study, due to our study is focused on breast and ovarian cancer which overwhelmingly affects female subjects.
Field-collected samples	No field collected samples were used in the study.
Ethics oversight	Experiments with mice were conducted under the approval of the Institutional Animal Care and Use Committee at The University of Texas MD Anderson Cancer Center, (protocol number: 00001250-RN01).

Note that full information on the approval of the study protocol must also be provided in the manuscript.

Flow Cytometry

Plots

Confirm that:

- [x] The axis labels state the marker and fluorochrome used (e.g. CD4-FITC).
- [x] The axis scales are clearly visible. Include numbers along axes only for bottom left plot of group (a 'group' is an analysis of identical markers).
- [x] All plots are contour plots with outliers or pseudocolor plots.
- [x] A numerical value for number of cells or percentage (with statistics) is provided.

Methodology

Sample preparation	Samples used in this study was U2OS DR-GFP cell line from cell culture, which was trypsinized and harvested to create cell suspensions in FACS buffer (2mM EDTA and 2% FBS in PBS). We provide the information in the Methods section.
Instrument	BD FACSCanto II cytometer
Software	BD FACSDiva 8.0.2 software and FlowJo 10.7.1 software
Cell population abundance	Cell population data were collected on a debris exclusion gate at the time of acquisition of FACSDiva software. A total of 50,000 cell events per sample was collected.
Gating strategy	Cell populations were gated on FSC/SSC for cell selection and debris exclusion. Next, a FSC-W/FSC-A plot was used to exclude the doublets. Single cells were further gated on AmCyan/FSC to select for living cells based on Ghost Dye™ Violet 510 staining. Live cells were further quantified by FITC to determine GFP positive cell populations. Gating strategy used for flow cytometric analysis is provided in Extended Data Fig. 3f and 3g.

☒ Tick this box to confirm that a figure exemplifying the gating strategy is provided in the Supplementary Information.
