# Supplementary figures and images for: Targeting the ALK–CDK9-Tyr19 kinase cascade sensitizes ovarian and breast tumors to PARP inhibition via destabilization of the P-TEFb complex
Source: Nat Cancer. 2022 Oct 17;3(10):1211–27. doi: 10.1038/s43018-022-00438-2 (PMC9586872; doi:10.1038/s43018-022-00438-2)

Fig 3a

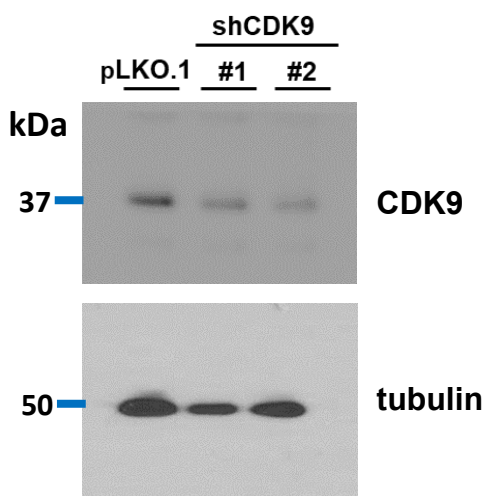

Fig 3e (left panel)

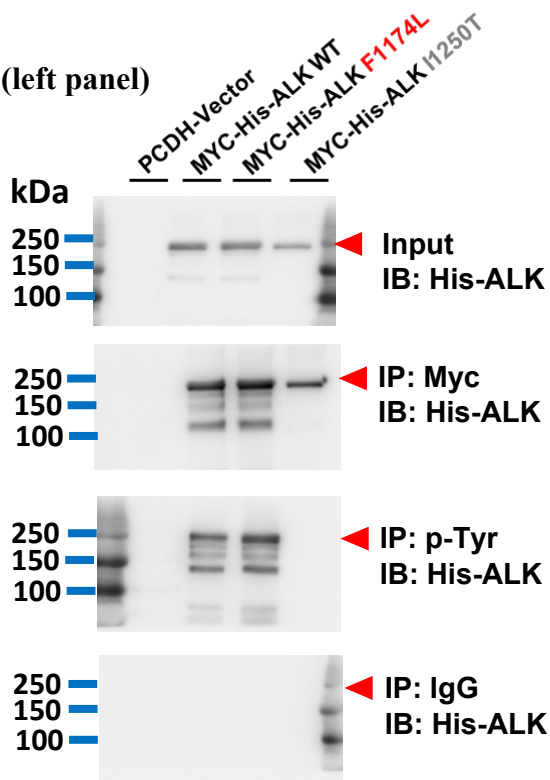

Fig 3e (right panel)

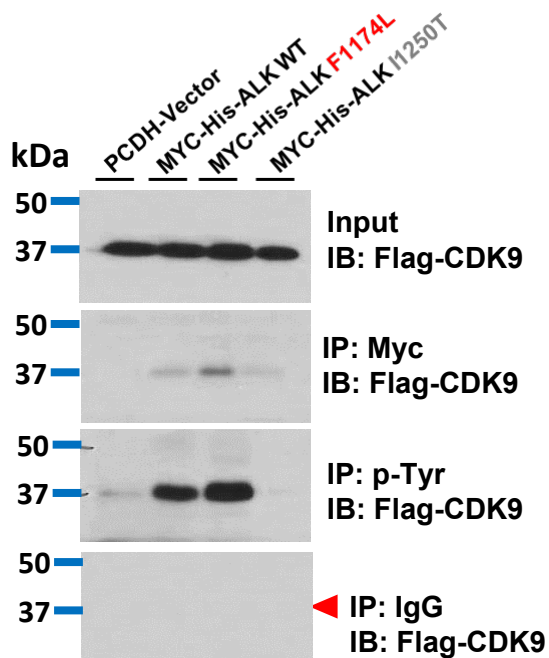

Fig 3f

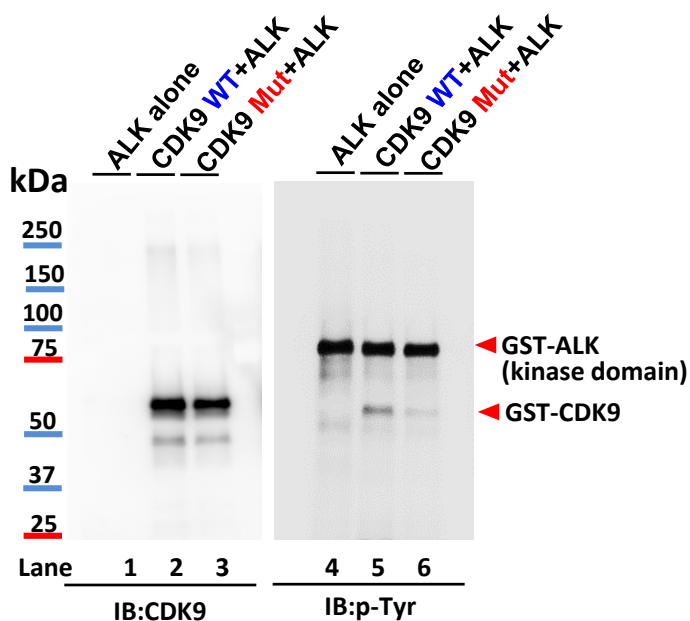

Fig 3g

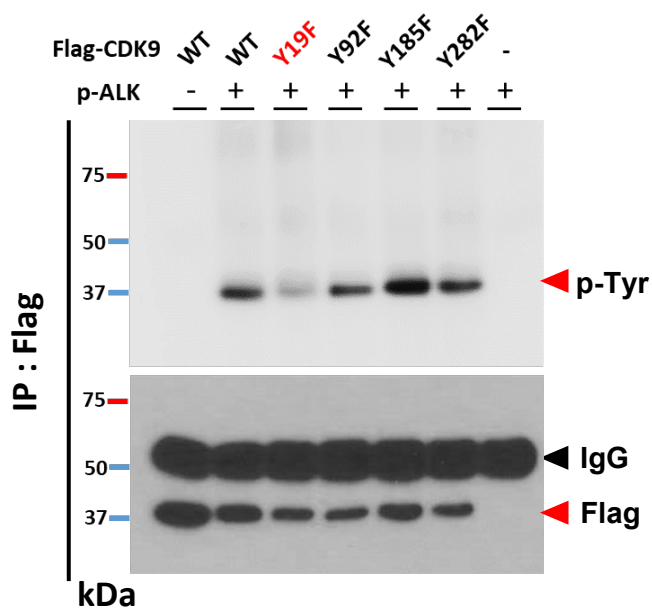

Fig 3i

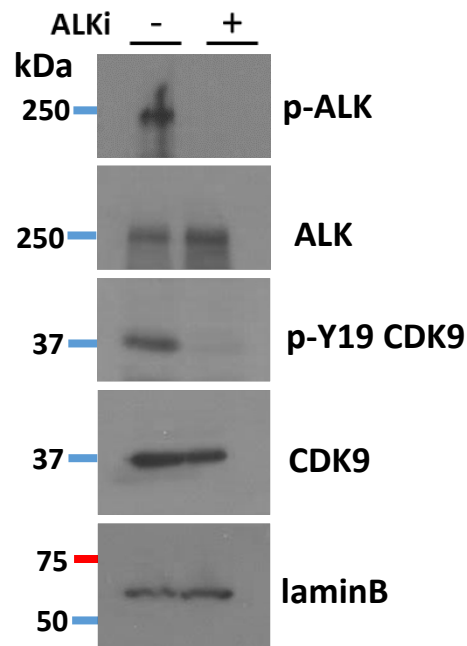

Fig 3h

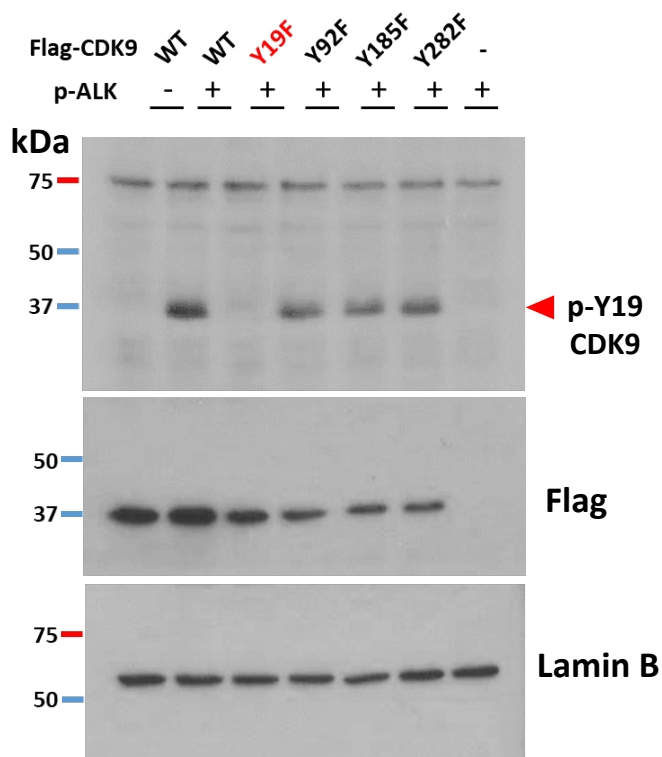

Fig 3j

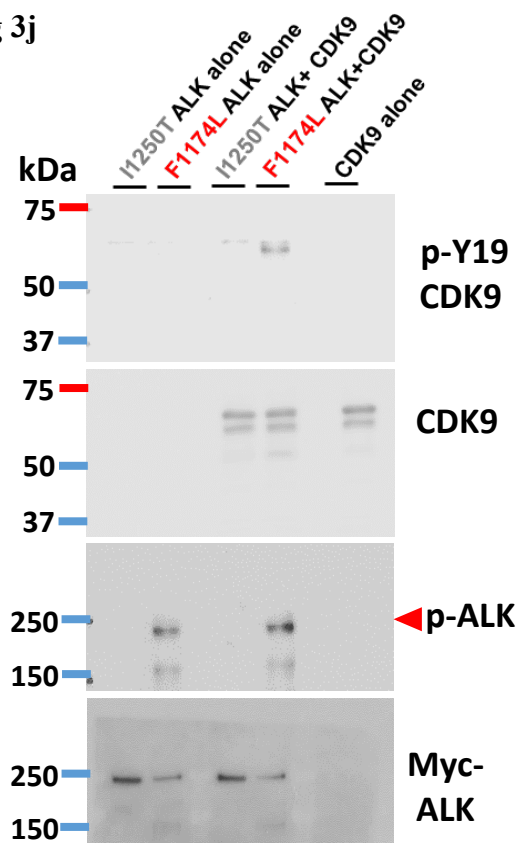

Supplement: Source Data Fig. 3 — Unprocessed western blots. [file 43018_2022_438_MOESM5_ESM.pdf]

Fig 5a

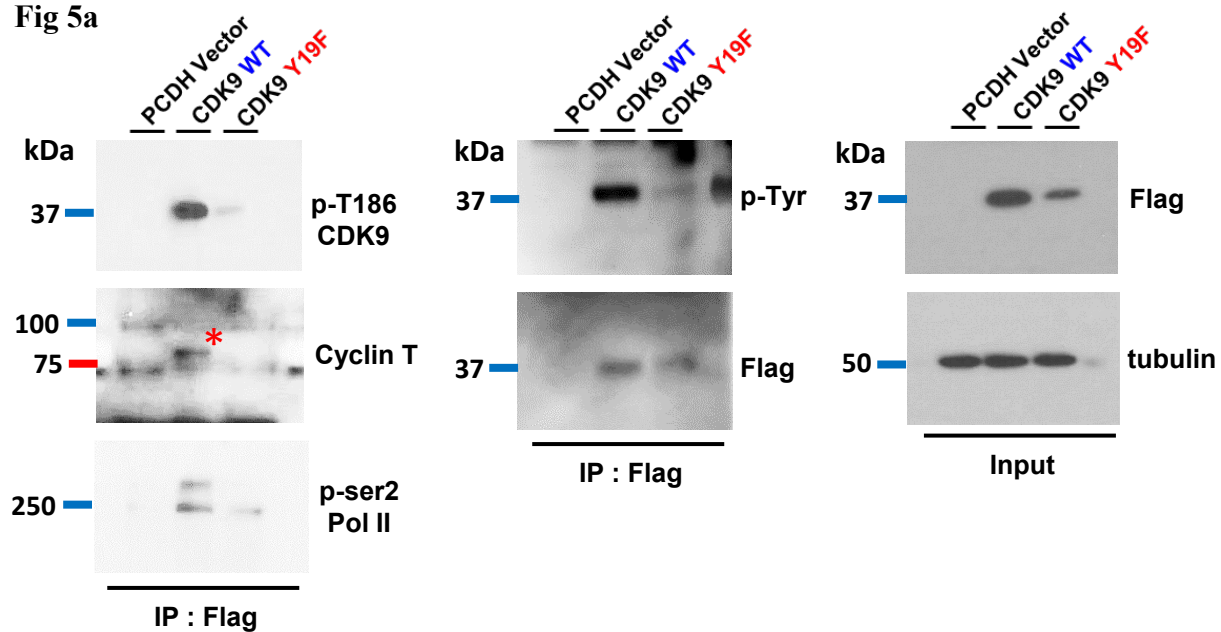

Fig 5b

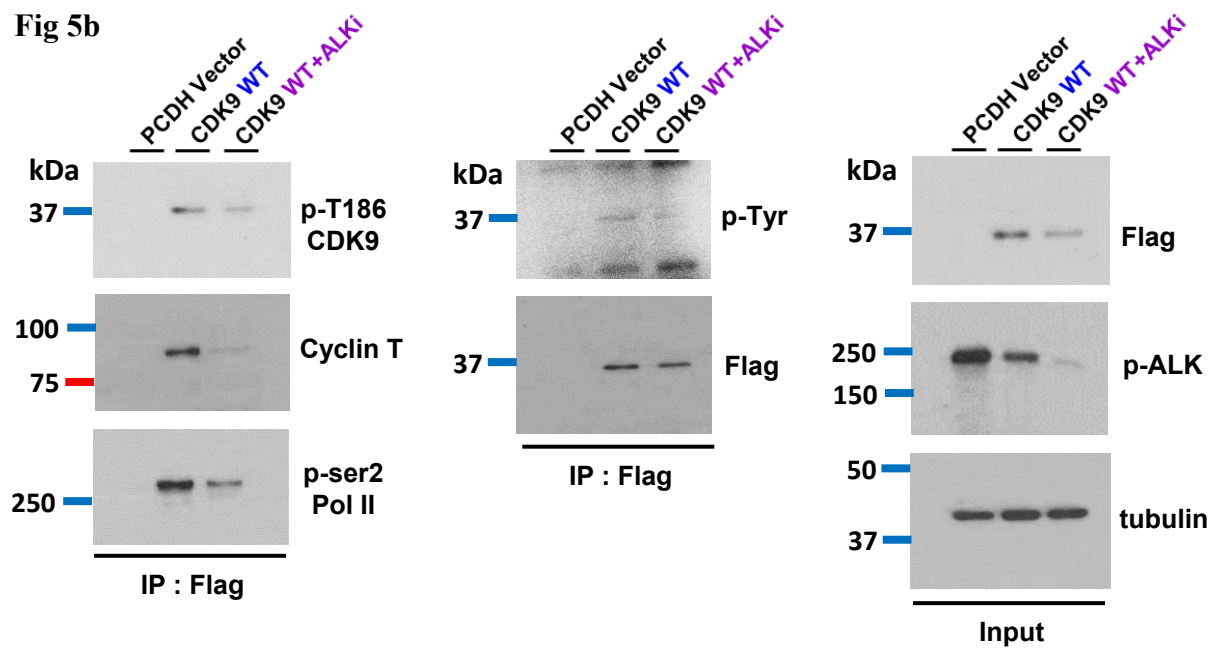

**Fig 5c**

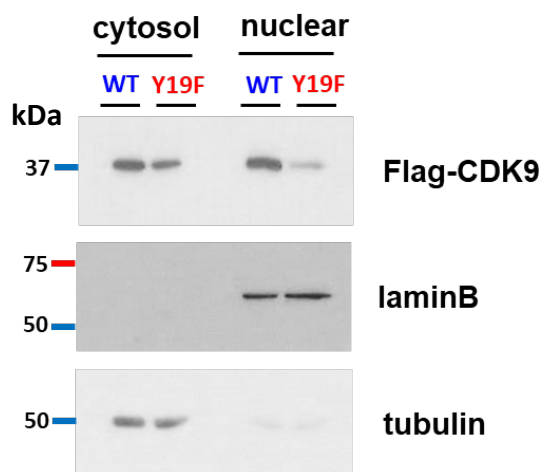

**Fig 5d**

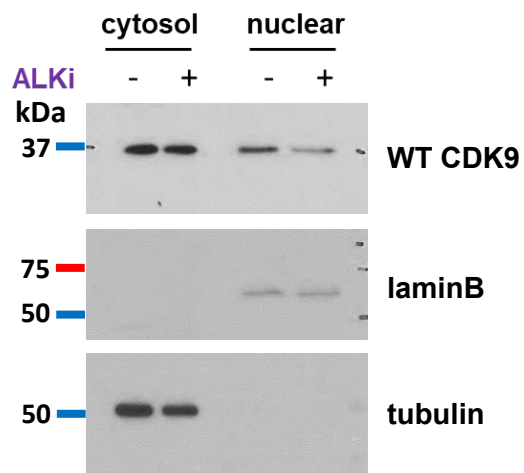

**Fig 5h**

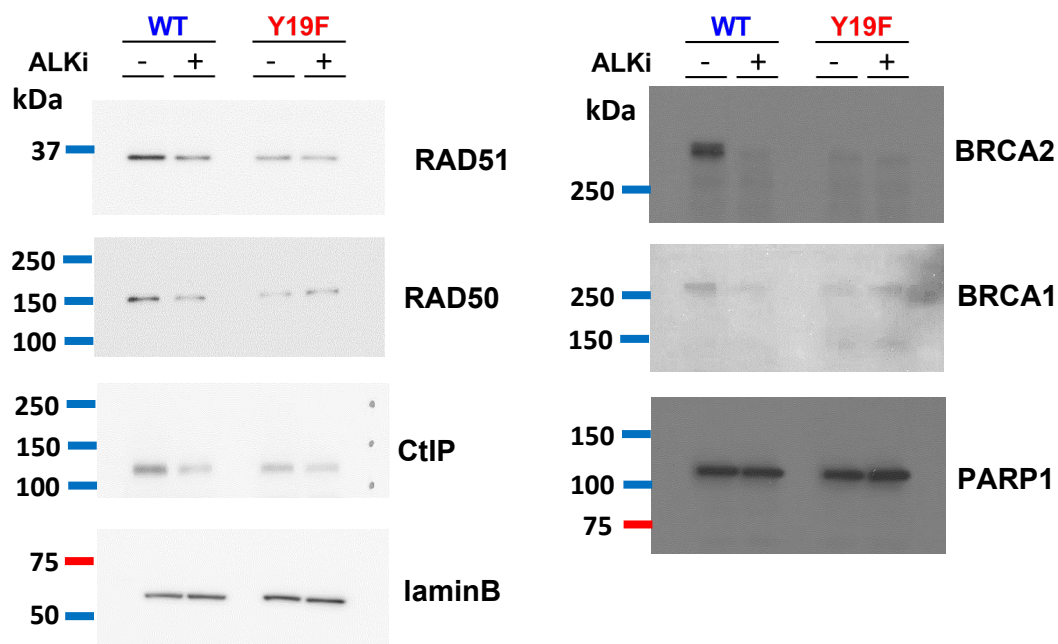

**Fig 5i**

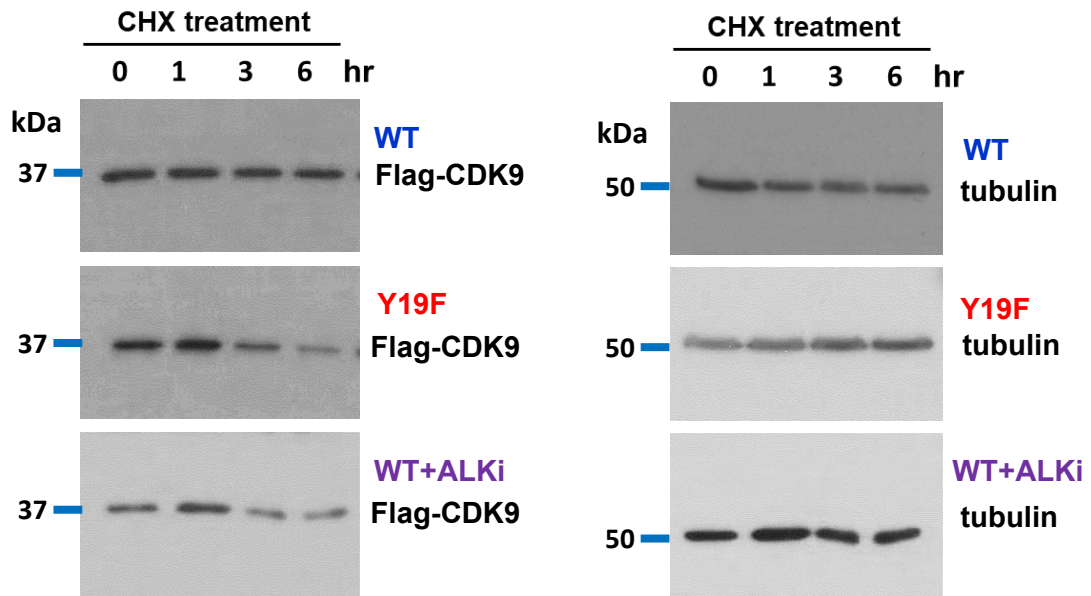

**Fig 5j**

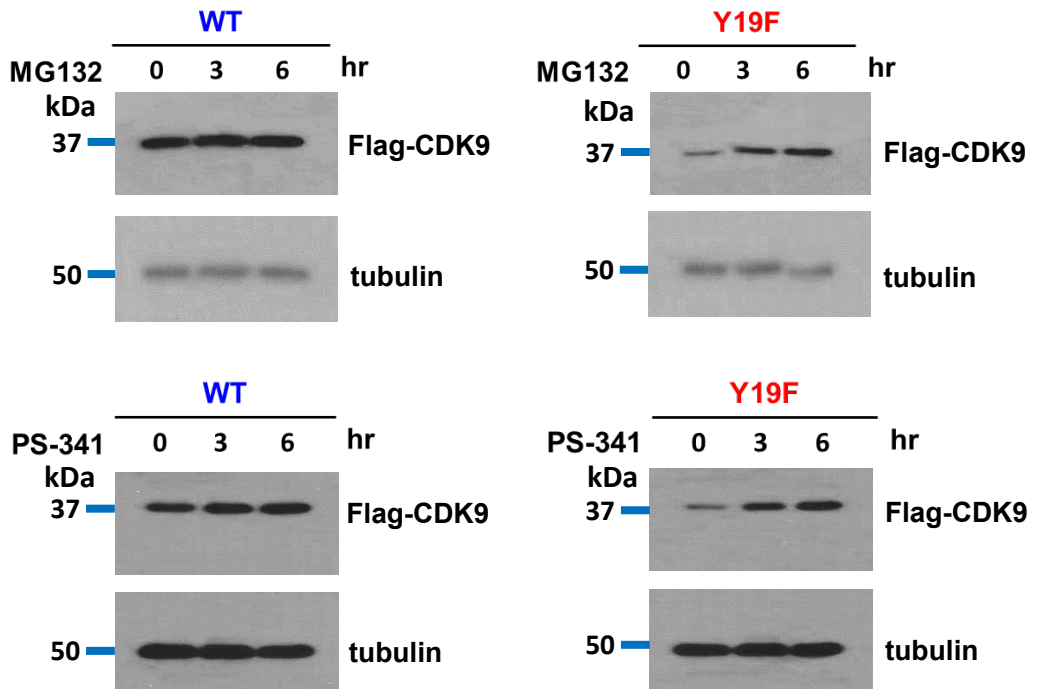

Fig 5k

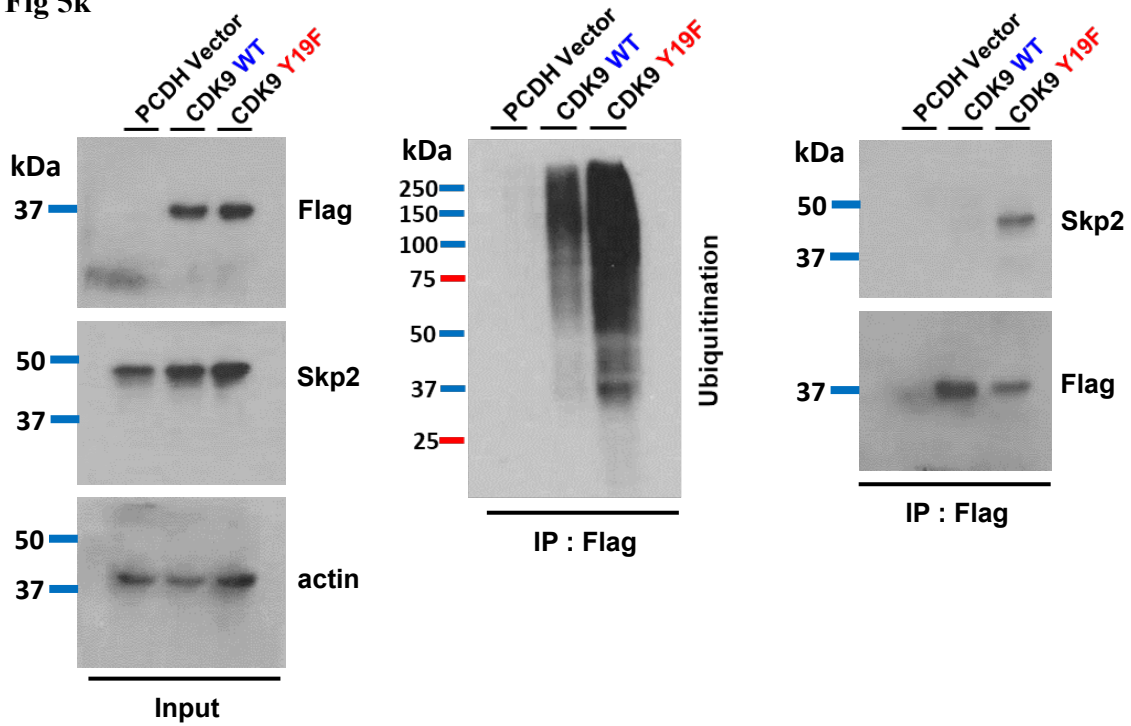

Fig 5l

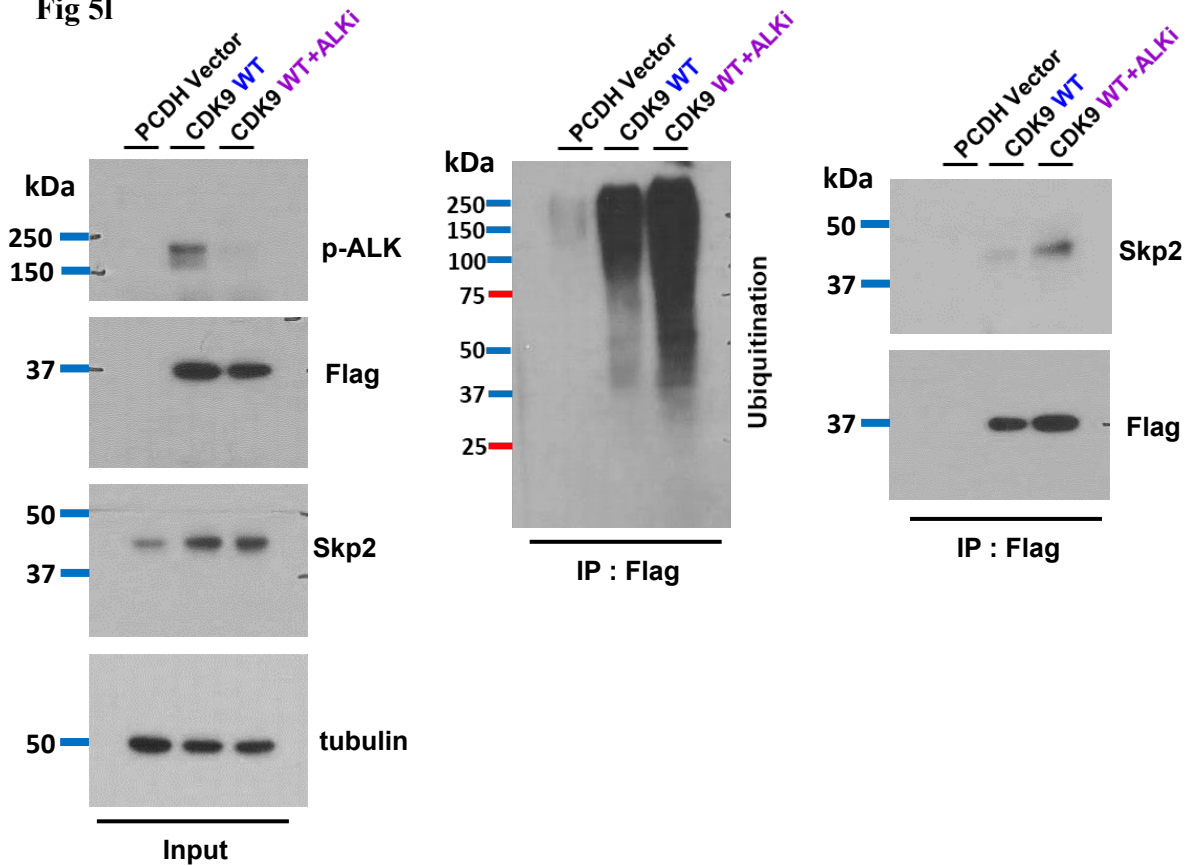

Supplement: Source Data Fig. 5 — Unprocessed western blots. [file 43018_2022_438_MOESM8_ESM.pdf]

Extended Data Fig 1a

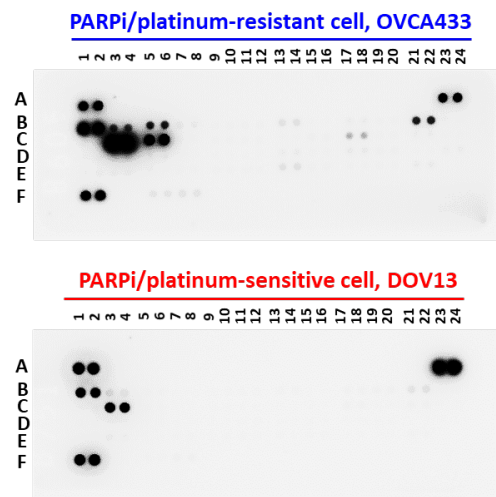

Extended Data Fig 1b

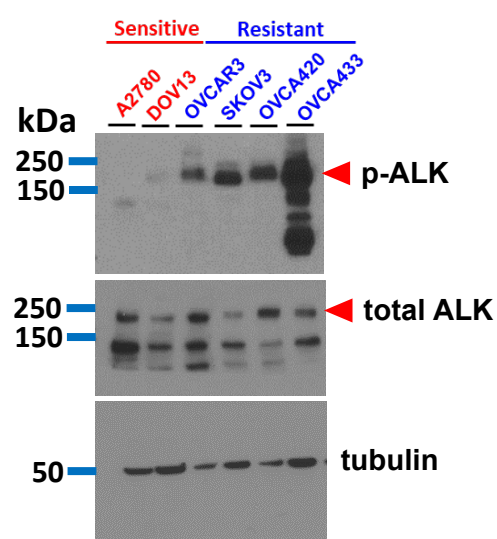

Extended Data Fig 1c

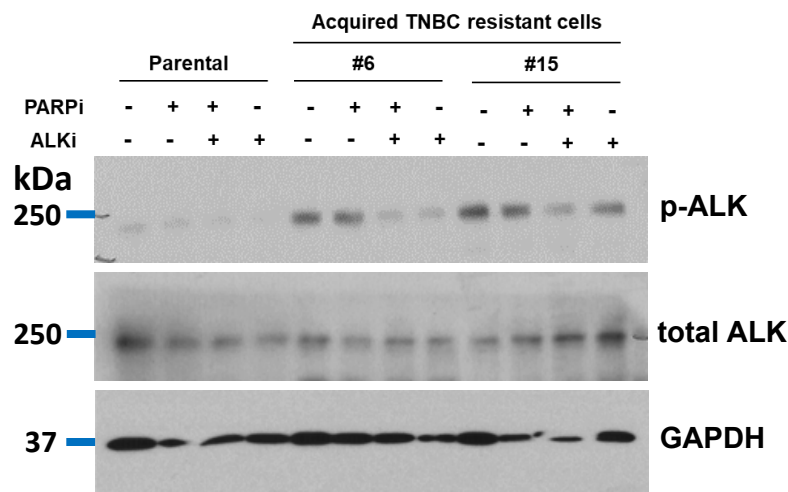

Supplement: Source Data Extended Data Fig. 1 — Unprocessed western blots. [file 43018_2022_438_MOESM11_ESM.pdf]

Extended Data Fig 4a

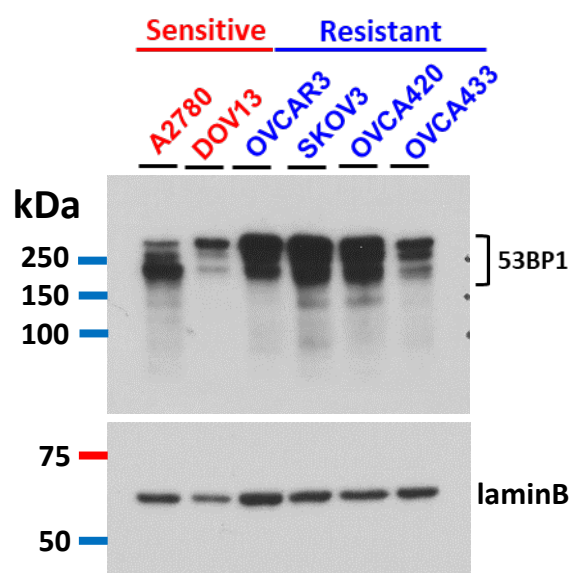

Extended Data Fig 4d

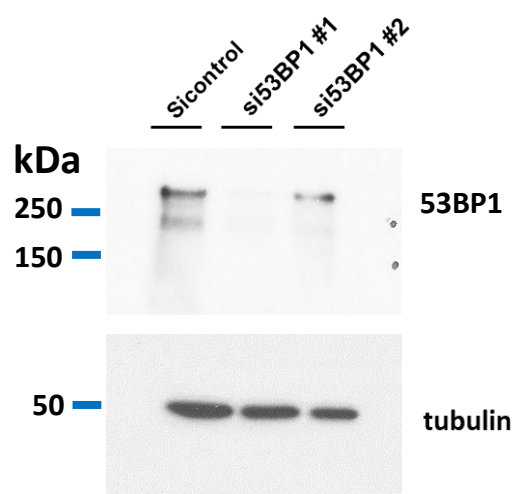

Supplement: Source Data Extended Data Fig. 4 — Unprocessed western blots. [file 43018_2022_438_MOESM14_ESM.pdf]

Extended Data Fig 5b

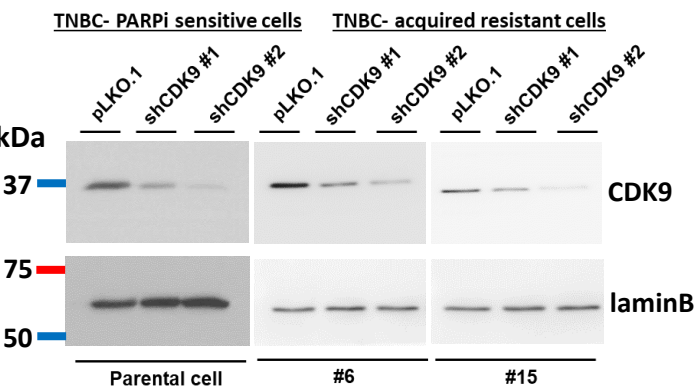

Extended Data Fig 5h

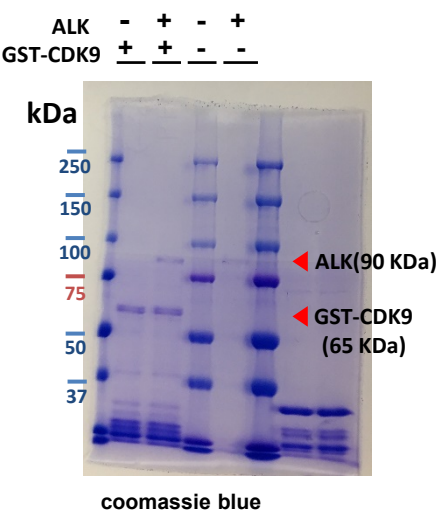

Extended Data Fig 5g

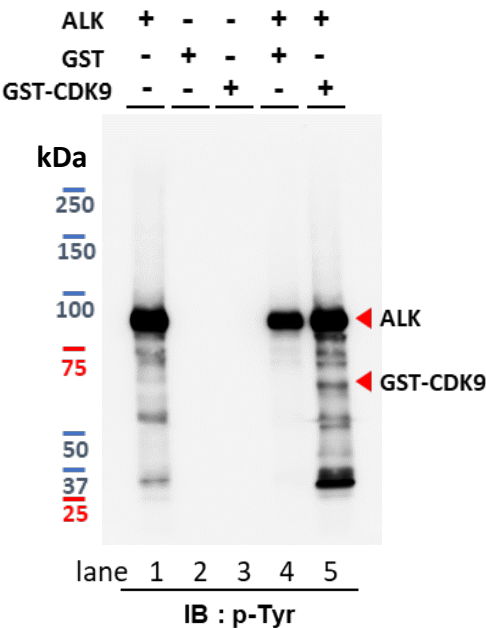

Extended Data Fig 5i

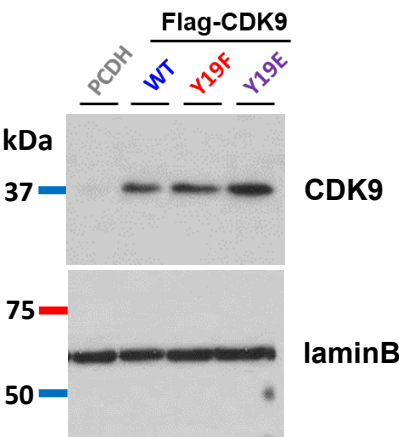

Supplement: Source Data Extended Data Fig. 5 — Unprocessed western blots. [file 43018_2022_438_MOESM16_ESM.pdf]

Extended Data Fig 7a

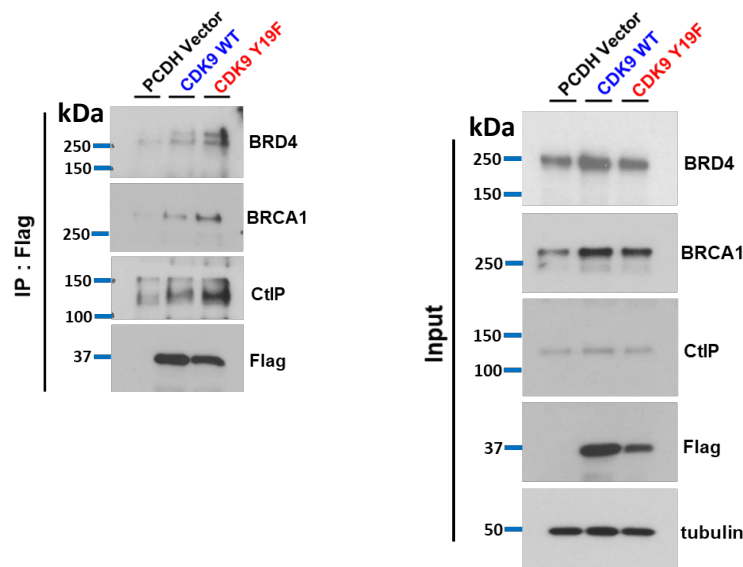

Extended Data Fig 7c

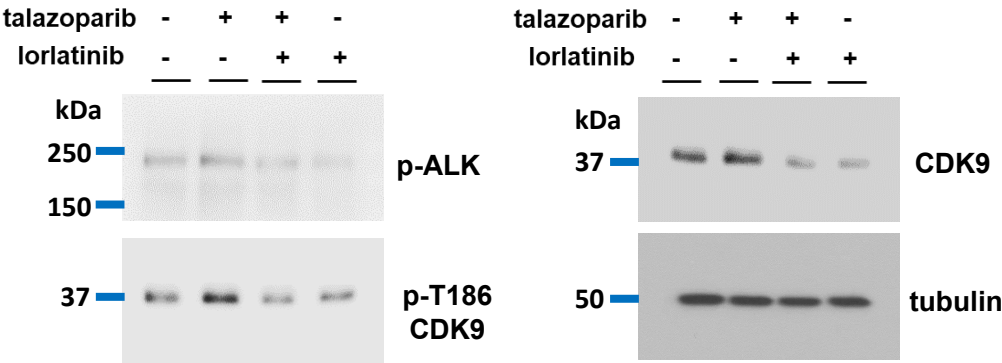

Supplement: Source Data Extended Data Fig. 7 — Unprocessed western blots. [file 43018_2022_438_MOESM18_ESM.pdf]
