# Supplementary material for: Targeting the ALK–CDK9-Tyr19 kinase cascade sensitizes ovarian and breast tumors to PARP inhibition via destabilization of the P-TEFb complex
Source: Nat Cancer. 2022 Oct 17;3(10):1211–27. doi: 10.1038/s43018-022-00438-2 (PMC9586872; doi:10.1038/s43018-022-00438-2)

Extended Data Fig 8b

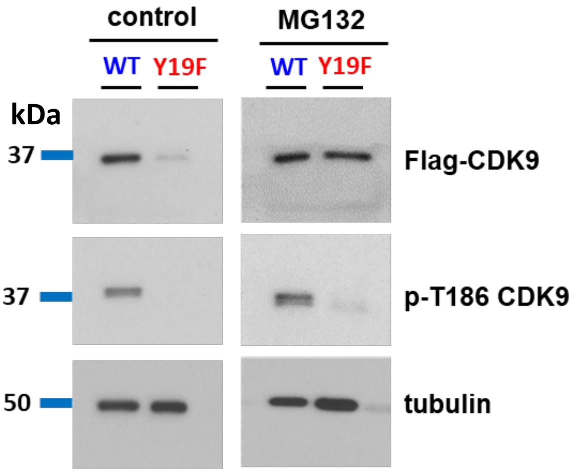

Extended Data Fig 8c

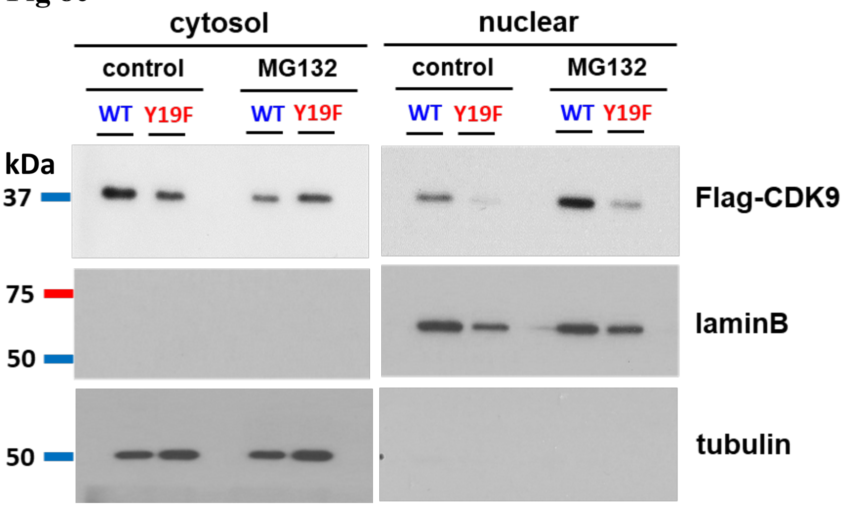

Extended Data Fig 8d

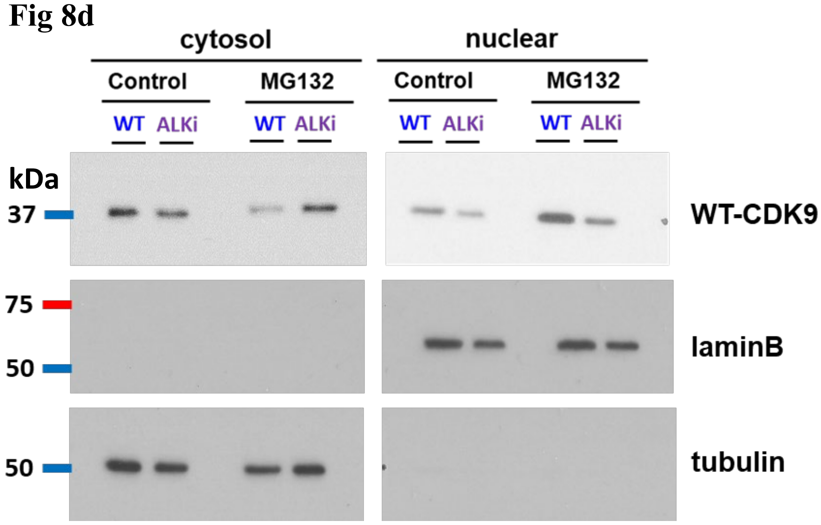

Supplement: Source Data Extended Data Fig. 8 — Unprocessed western blots. [file 43018_2022_438_MOESM20_ESM.pdf]
